# Ni‐Ion‐Chelating Strategy for Mitigating the Deterioration of Li‐Ion Batteries with Nickel‐Rich Cathodes

**DOI:** 10.1002/advs.202205918

**Published:** 2022-12-16

**Authors:** Seon Yeong Park, Sewon Park, Hyeong Yong Lim, Moonsu Yoon, Jeong‐Hee Choi, Sang Kyu Kwak, Sung You Hong, Nam‐Soon Choi

**Affiliations:** ^1^ Battery R&D Center SK On 325, Expo‐ro, Yuseong‐gu Daejeon 34124 Republic of Korea; ^2^ School of Energy and Chemical Engineering Ulsan National Institute of Science and Technology (UNIST) 50 UNIST‐gil Ulsan 44919 Republic of Korea; ^3^ Department of Nuclear Science and Engineering Massachusetts Institute of Technology Cambridge MA 02139 United States; ^4^ Next Generation Battery Research Center Korea Electrotechnology Research Institute 12 Jeongiui‐gil, Seongsan‐gu, Changwon‐si Gyeongsangnam‐do 51543 Republic of Korea; ^5^ Electro‐Functionality Materials Engineering University of Science and Technology (UST) 217 Gajeong‐ro, Yuseong‐gu Daejeon 34113 Republic of Korea; ^6^ Department of Chemical and Biological Engineering Korea University 145 Anam‐ro, Seongbuk‐gu Seoul 02841 Republic of Korea; ^7^ Department of Chemistry Ulsan National Institute of Science and Technology (UNIST) 50 UNIST‐gil Ulsan 44919 Republic of Korea; ^8^ Department of Chemical and Biomolecular Engineering Korea Advanced Institute of Science and Technology (KAIST) 291 Daehak‐ro, Yuseong‐gu Daejeon 34141 Republic of Korea

**Keywords:** chelating agents, electrolyte additives, lithium‐ion batteries, nickel‐rich cathodes, transition metal dissolution

## Abstract

Ni‐rich cathodes are the most promising candidates for realizing high‐energy‐density Li‐ion batteries. However, the high‐valence Ni^4+^ ions formed in highly delithiated states are prone to reduction to lower valence states, such as Ni^3+^ and Ni^2+^, which may cause lattice oxygen loss, cation mixing, and Ni ion dissolution. Further, LiPF_6_, a key salt in commercialized electrolytes, undergoes hydrolysis to produce acidic compounds, which accelerate Ni‐ion dissolution and the interfacial deterioration of the Ni‐rich cathode. Dissolved Ni ions migrate and deposit on the surface of the graphite anode, causing continuous electrolyte decomposition and threatening battery safety by forming Li dendrites on the anode. Herein, 1,2‐bis(diphenylphosphino)ethane (DPPE) chelates Ni ions dissolved from the Ni‐rich cathode using bidentate phosphine moieties and alleviates LiPF_6_ hydrolysis via complexation with PF_5_. Further, DPPE reduces the generation of corrosive HF and HPO_2_F_2_ substantially compared to the amounts observed using trimethyl phosphite and tris(trimethylsilyl) phosphite, which are HF‐scavenging additives. Li‐ion cells with Ni‐rich cathodes and graphite anodes containing DPPE exhibit remarkable discharge capacity retentions of 83.4%, with high Coulombic efficiencies of >99.99% after 300 cycles at 45 °C. The results of this study will promote the development of electrolyte additives.

## Graphical abstract

1,2‐Bis(diphenylphosphino)ethane (DPPE), as an electrolyte additive, chelates Ni^2+^ dissolved from the LiNi_0.85_Co_0.1_Mn_0.05_O_2_ cathode and suppresses the electrodeposition of Ni on the graphite anode, which prevents the lithiation of graphite and induces the overpotential of lithiation. DPPE deactivates Lewis acidic PF_5_, preventing HF generation. The decreased HF content mitigates Ni dissolution from the cathode and alleviates structural deterioration of the cathode.

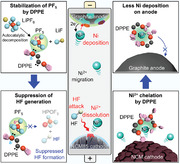

## 1 Introduction

The proliferation of electric vehicles has increased exponentially over the past few years, and the ban on petrol and diesel vehicles in European nations has caused automakers to switch their primary focus toward electric vehicles.^[^ ^1^, ^2^ ^]^ Engines, which are the most critical components of automobiles, have been replaced by motors with batteries; specifically, batteries containing Ni‐rich cathodes are essential in ensuring high performances, for example, in extending the mileage of electric vehicles.^[^ ^3^, ^4^ ^]^ However, high‐valence Ni^4+^, which is formed in a highly charged state in such batteries, is prone to reduction to Ni^3+^ and Ni^2+^, resulting in oxygen loss and cation mixing. In addition, residual Li species, such as LiOH and Li_2_CO_3_, induce parasitic reactions in electrolytes.^[^ ^5^, ^6^ ^]^ Furthermore, Ni^2+^ dissolved from Ni‐rich cathodes by acidic compounds, such as HF formed by LiPF_6_ hydrolysis in the electrolyte, induces structural deterioration by forming an inactive rock‐salt phase and the loss of the Li storage sites of the cathode.^[^ ^7^, ^8^, ^9^ ^]^ Further, transition metals electrodeposited at the anode surface via the dissolution–migration–deposition of transition metal ions (TM‐DMD) hinder the intercalation of Li^+^ within the anode structure, catalyze undesirable electrolyte decomposition reactions,^[^ ^10^, ^11^ ^]^ and act as solid–electrolyte interphase (SEI) components. The electrodeposited transition metals also increase the probability of the formation of dendritic Li, which threatens battery safety.^[^ ^12^ ^]^ Thus far, surface coating of the cathode^[^ ^13^, ^14^, ^15^ ^]^ and forming a protective film on the cathode using functional electrolyte additives^[^ ^16^, ^17^, ^18^, ^19^ ^]^ have been proposed as viable solutions to minimize transition‐metal‐ion dissolution from LiNi_0.85_Co_0.1_Mn_0.05_O_2_ (NCM85) cathodes. However, Ni^2+^, which is similar to Li^+^ in size,^[^ ^20^, ^21^ ^]^ can easily penetrate the surface coating layers and cathode protective films because they should guarantee facile Li^+^ transport while blocking electron transfer to prevent electrolyte decomposition.^[^ ^22^ ^]^ Electrolyte additives that suppress HF generation or scavenge HF^[^ ^23^, ^24^ ^]^ do not completely remove HF, and thus, the cells exhibit Ni‐dissolution‐ and deposition‐related problems.

Chelation of the dissolved transition metal ions may prevent electrodeposition on the surface of the anode.^[^ ^25^, ^26^, ^27^, ^28^ ^]^ However, transition‐metal chelating agents can hardly be applied as electrolyte additives because they decompose electrochemically at the electrodes, resulting in shortened battery lifespans. Studies regarding chemically active separators^[^ ^25^, ^27^, ^29^, ^30^ ^]^ with insoluble bipyridine (C—N) ligands and gel polymer electrolytes based on polymer matrices containing pyrrolidone (C—N—C=O) moieties^[^ ^28^ ^]^ as chelating functional groups have been conducted in efforts to avoid undesirable decomposition of the chelating agents at electrodes. Nevertheless, the incorporation of a chelating agent into the electrolyte without additional processing is clearly a more efficient method of capturing Ni ions from scalability and techno‐economic standpoints. Further, the microquantity of chelating agent as an electrolyte additive does not cause significant changes in the rheological, chemical, or electrochemical properties of the electrolyte, which may increase the cell impedance.

With the aim of enhancing cell performance, herein, we report the use of a tricoordinate phosphorous compound, 1,2‐bis(diphenylphosphino)ethane (DPPE), to provide effective donor ligands that are capable of forming complexes with Ni^2+^ dissolved in electrolytes, thereby preventing the electrodeposition of Ni^2+^ on the anode surface. Further, DPPE as a Lewis base additive can deactivate Lewis acidic PF_5_, which can generate corrosive HF, mitigate the damage of the SEI and cathode electrolyte interface (CEI), and alleviate PF_5_‐driven electrolyte solvent decomposition.

## 2 Results and Discussion

### 2.1 DPPE Chelation of the Ni Ions Dissolved From a Ni‐Rich Cathode

**Figure 1:**
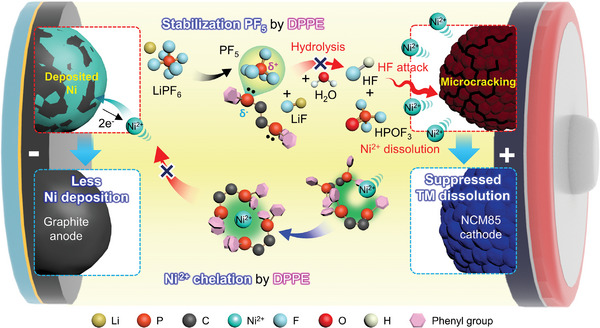
Schematic diagram of DPPE chelating Ni^2+^, stabilizing PF_5_, and preventing TM‐DMD initiated by LiPF_6_ hydrolysis.

**Figure 2:**
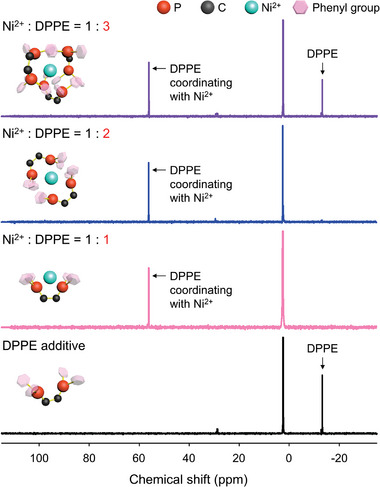
^31^P NMR spectra of the solutions containing Ni(TFSI)_2_ and DPPE in different ratios with an internal reference (trimethyl phosphate, 2.47 ppm).

Unlike diamine, bipyridine, or pyrrolidone compounds within microporous separators or gel polymer electrolytes,^[^ ^30^, ^31^ ^]^ DPPE contains phosphine groups, which may form a bidentate chelation complex with Ni^2+^ (**Figure** **1**).^[^ ^32^ ^]^ The electron‐rich P atoms of the phosphine groups may trap Ni^2+^ effectively.^[^ ^33^ ^]^ Ni^2+^ is a 16‐electron species, which violates the 18‐electron rule because low‐spin d^8^ Ni^2+^ is square‐planar. With the eight electrons in the 3d orbital, eight electrons are required to accumulate the 16 electrons. Because the 2 P atoms of the phosphine groups bear unshared electron pairs, two DPPE molecules, with four unshared electron pairs, may form a 16‐electron complex with Ni^2+^. To explore the Ni^2+^‐chelation ability of DPPE and coordination structure of DPPE with Ni^2+^, we added 2.5, 5.0, or 7.5 mm DPPE to solutions comprising 2.5 mm nickel bis(trifluoromethylsulfonyl)imide) (Ni(TFSI)_2_) and ethylene carbonate (EC)/dimethyl carbonate (DMC)/ethyl methyl carbonate (EMC) (1/2/2, v/v/v). Pure DPPE (2.5 mm) in EC/EMC/DMC (1/2/2, v/v/v) forms a transparent, colorless solution (Figure S1a,b, Supporting Information). Conversely, 2.5, 5.0, or 7.5 mm DPPE with 2.5 mm Ni(TFSI)_2_, with 1:1, 1:2, and 1:3 equivalent ratios of Ni^2+^ and DPPE, respectively, form transparent yellowish solutions due to the square‐planar structures of the coordinated Ni^2+^ and chelating agent (Figure S1c–e, Supporting Information).^[^ ^34^ ^]^ The ^31^P NMR spectrum of the solution with a 1:1 equivalent ratio of Ni^2+^ and DPPE exhibits no signal representing phosphine at −13.2 ppm and a new signal at 56.1 ppm, which is attributed to the [Ni(DPPE)_2_]^2+^ complex (**Figure** **2**).^[^ ^35^ ^]^

The spectrum of the solution with the 1:2 equivalent ratio of Ni^2+^ and DPPE displays a more intense signal representing [Ni(DPPE)_2_]^2+^, and the spectrum of the solution with the 1:3 equivalent ratio exhibits a signal representing [Ni(DPPE)_2_]^2+^ with a similar integrated area at 56.1 ppm (Table S1, Supporting Information). Notably, the peak at −13.2 ppm, which represents DPPE with no interactions with Ni^2+^, is observed in the spectrum of the solution with the 1:3 equivalent ratio of Ni^2+^ and DPPE. This suggests that Ni^2+^ coordinates with 2 DPPE molecules to form Ni(DPPE)_2_^2+^. Furthermore, as the strong affinity of the bidentate phosphine toward Ni^2+^ may extract Ni ions from the NCM85 cathode, leading to a reduction in Li storage capacity, incorporating the optimal content of DPPE should be considered. Steric hindrance due to the bulky phenyl groups of DPPE should prevent the direct binding of the phosphine groups with Ni ions at the surface of the NCM85 cathode.^[^ ^36^ ^]^ Further, the phenyl groups, as inductively electron‐withdrawing groups,^[^ ^37^ ^]^ may optimize the binding force of the bidentate phosphine with Ni ions by tuning the electronegativity of trivalent P.

**Figure 3:**
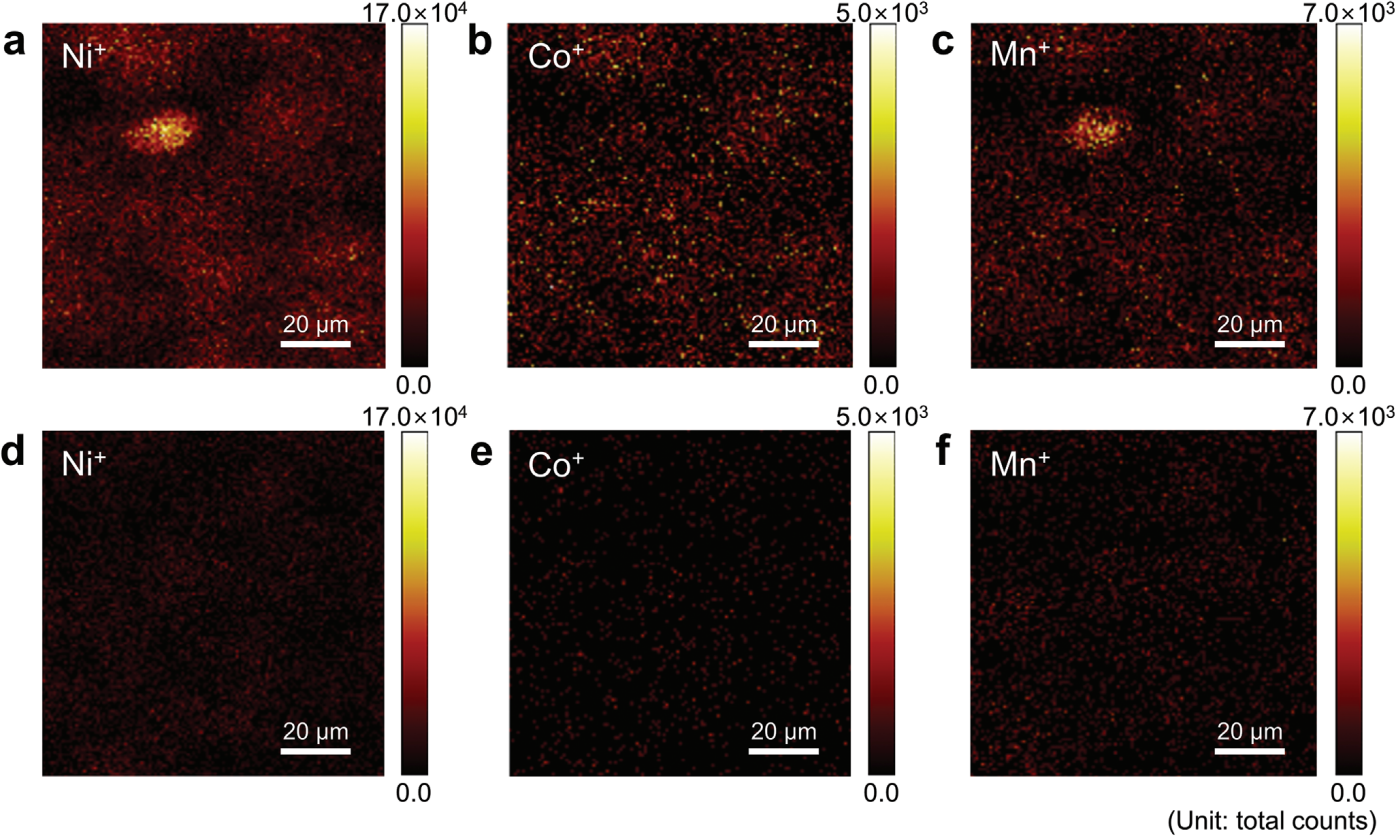
TOF‐SIMS images of Ni^+^, Co^+^, and Mn^+^ on graphite anodes retrieved from full cells cycled at 45 °C with the a–c) baseline or d–f) DPPE‐containing electrolyte.

To confirm the optimization of the binding force of the phenyl group in the interaction between the bidentate phosphine ligands and Ni ions, delithiated NCM85 cathodes were stored in electrolytes with DPPE or bis(dimethylphosphino)ethane (DMPE), with less bulky methyl groups, at 60 °C for 24 h. Inductively coupled plasma‐optical emission spectroscopy (ICP‐OES) reveals that the electrolyte with DMPE exhibits a dissolved Ni^2+^ ion concentration (3.60 ppm) that is fourfold higher compared with the baseline electrolyte (1.15 m LiPF_6_ in EC/DMC/EMC (1/2/2, v/v/v) with 1 wt.% vinylene carbonate (VC), 0.83 ppm, Table S2, Supporting Information). In contrast, the electrolyte with DPPE exhibits a reduced concentration of dissolved Ni^2+^ ions (0.37 ppm). This suggests that methyl groups, with electron‐donating characters, increase the electron densities of the P atoms of DMPE, which thus severely leaches Ni ions from the NCM85 cathode. According to molecular electrostatic potential mapping using density functional theory (DFT) calculations, the P atoms of DMPE display strong nucleophilicities compared to those of the P atoms of DPPE (Figure S2a, Supporting Information). Furthermore, DMPE exhibits surface‐accessible nucleophilic P, whereas the P of DPPE is inaccessible due to the bulky phenyl groups. Based on the binding energies with Ni^2+^ (Figure S2b, Supporting Information), a moderate interaction between Ni^2+^ and DPPE is possible, whereas DMPE is likely to strongly chelate Ni^2+^, leading to undesired leaching from the NCM85 cathode. To study the Ni^2+^ chelation of DPPE in an NCM85/graphite full cell, cells were cycled with electrolytes containing 0.188 mm Ni(TFSI)_2_ with and without 0.376 mm DPPE (1:2 equivalent ratio). The graphite anode used with 0.188 mm Ni(TFSI)_2_ + 0.376 mm DPPE displays an extremely weak Ni^+^ signal, which is strong when using the electrolyte with 0.188 mm Ni(TFSI)_2_ (Figure S3, Supporting Information). This suggests that DPPE hinders Ni deposition on the graphite anode via complexation with Ni^2+^. Moreover, the addition of DPPE to the electrolyte reduces the interfacial resistances of the NCM85/graphite full cells after precycling, which is likely because the Ni deposition‐induced resistance is decreased (Figure S4, Supporting Information). Time‐of‐flight secondary ion mass spectrometry (TOF‐SIMS) imaging of the graphite anode from the full cell after 300 cycles at 45 °C in the baseline electrolyte reveals strong Ni^+^, Co^+^_,_ and Mn^+^ signals. This is because of the accumulation of transition metals on the graphite anode via TM‐DMD (**Figure** **3**). Conversely, transition metal signals are not apparent on the graphite anode cycled in the 0.1 wt.% DPPE‐containing electrolyte. The favorable effect of DPPE on TM‐DMD was confirmed using ICP‐OES, revealing a reduced Ni content (62.9 ppm, baseline electrolyte: 154 ppm) on the graphite anode after DPPE addition (Table S3, Supporting Information). However, the graphite anode cycled with 0.1 wt.% DMPE, with an immoderate affinity toward Ni ions, exhibits a similar Ni content to that of the graphite anode cycled in the baseline electrolyte. Therefore, DPPE, with a moderate affinity toward Ni ions, effectively chelate Ni^2+^ dissolved from the NCM85 cathode and suppresses Ni deposition on the anode surface, alleviating the degradation in cell performance. Based on the similarity in Ni contents detected on graphite anodes with 0.1 wt.% and 0.5 wt.% DPPE (Table S3, Supporting Information) and calculations considering the coordination of Ni^2+^ ions with two DPPE molecules (S1 and S2, Supporting Information), 0.1% DPPE in the electrolyte may be assumed to be sufficient for scavenging Ni^2+^ ions dissolved from NCM85 cathode. Moreover, DPPE chelates Ni^2+^, Mn^2+^, and Co^2+^ ions in the electrolyte; thus, transition metal (Ni, Co, Mn) deposition on the graphite anodes is greatly mitigated (Table S4, Supporting Information). The optimal DPPE content was confirmed to be 0.1 wt.% by comparing the cycle performance of a full cell with this electrolyte with that of a full cell with 0.5 wt.% DPPE‐containing electrolyte over 300 cycles at 25 and 45 °C (Figure S5, Supporting Information).

### 2.2 DPPE Stabilization of PF_5_ in an LiPF_6_‐Containing Electrolyte

**Figure 4:**
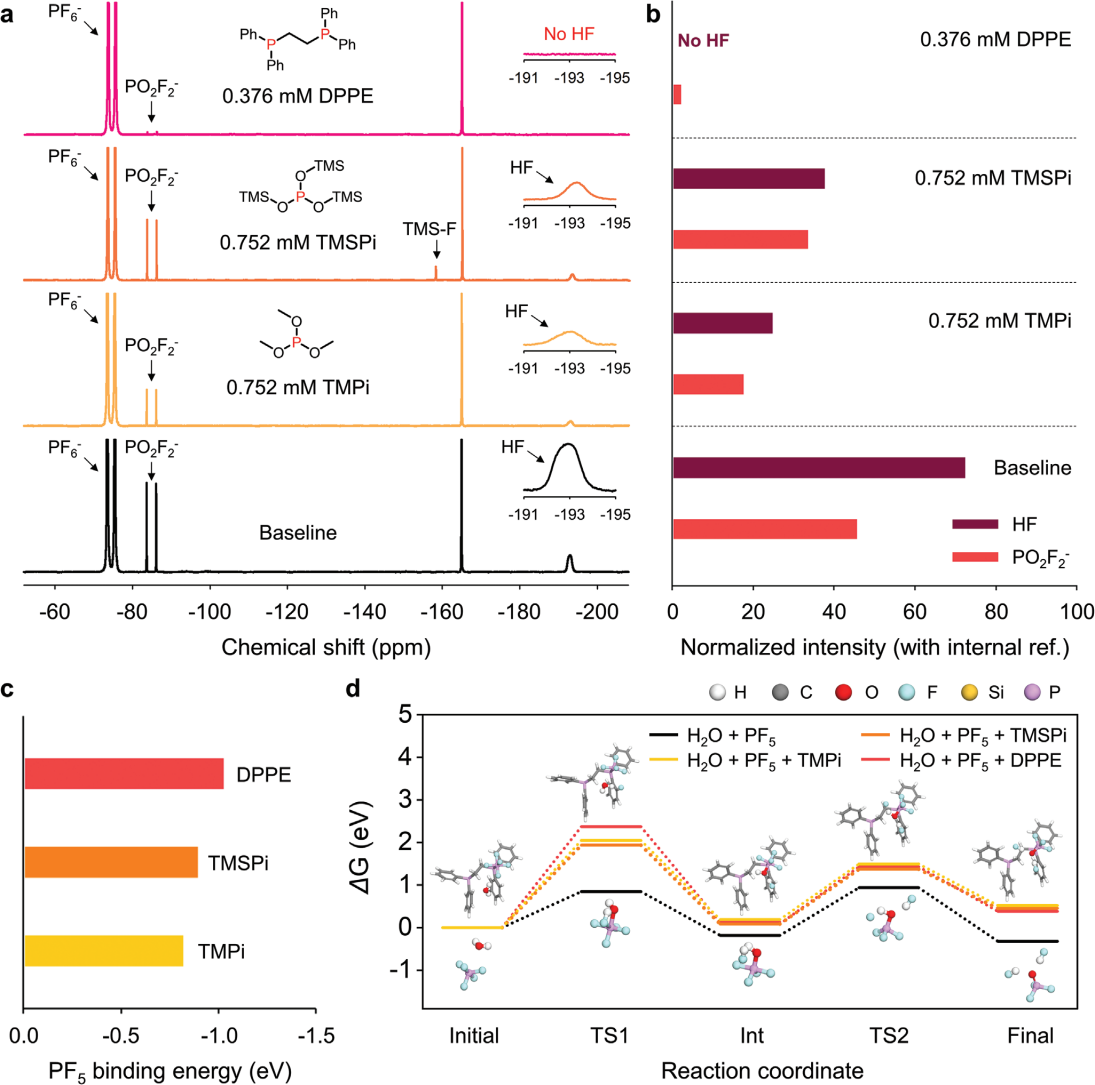
a) ^19^F NMR spectra of the baseline, TMPi‐containing, TMSPi‐containing, and DPPE‐containing electrolytes after storage for 1 d at 25 °C. b) Integrated areas of the signals representing HF and PO_2_F_2_^−^ in the ^19^F NMR spectra normalized using an internal reference (C_6_F_6_). c) Binding energies of PF_5_ with TMPi, TMSPi, and DPPE. d) Relative Gibbs free energies (ΔG) of the hydrolyses of PF_5_ with and without different additives. TS1: transition state 1, Int: intermediate step, TS2: transition state 2.

LiPF_6_‐containing electrolytes display undesirable hydrolysis of LiPF_6_, producing harmful acidic species such as HF and HPO_2_F_2_, which negatively affect battery materials.^[^ ^38^, ^39^, ^40^, ^41^ ^]^ HF leads to the degradation of the protective interfacial layers on electrodes and the decomposition of electrolyte solvent molecules to form gaseous compounds, such as CO_2_ (EC + H^+^ → ^+^H_2_O‐CH_2_CH_2_‐OCO_2_H → HO‐CH_2_CH_2_‐OH + CO_2_ + H^+^), resulting in electrolyte depletion and battery swelling.^[^ ^42^, ^43^ ^]^ Further, the proton of HF reacts with lattice oxygen to form H_2_O, and Jahn–Teller‐active Ni^3+^ undergoes disproportionation to Ni^2+^ and Ni^4+^, with Ni^2+^ dissolution in the electrolyte promoting a severe phase transition.^[^ ^44^, ^45^ ^]^ To suppress the formation of acidic compounds in LiPF_6_‐based electrolytes, various strategies, such as the deactivation of PF_5_ to hinder hydrolysis^[^ ^46^, ^47^, ^48^ ^]^ and the scavenging of generated acidic compounds,^[^ ^49^, ^50^ ^]^ have been proposed. The phosphite moiety, with an electron‐donating character, forms a complex with Lewis acidic PF_5_ and a silyl ether group with a high uptake capacity for the F of HF via the formation of pentavalent Si—F bonds.^[^ ^51^ ^]^ Although tris(trimethylsilyl) phosphite (TMSPi), which is one of the HF scavenging additives, stabilizes PF_5_,^[^ ^52^, ^53^ ^]^ the trimethyl silyl groups degrade the performance of the Ni‐rich cathode because of parasitic reactions with residual Li compounds, such as LiOH.^[^ ^54^ ^]^ As DPPE bears two phosphine groups with electron‐rich trivalent P, it should inhibit hydrolysis to prevent the formation of acidic compounds, including HF, by capturing PF_5_. To study the PF_5_ stabilization effect of DPPE, 1000 ppm of H_2_O was added to the baseline electrolyte with and without 0.376 mm DPPE, and ^19^F NMR spectroscopy of each electrolyte was conducted after storage at 25 °C for 24 h (**Figure** **4**a,b). To measure the PF_5_ stabilizing capacity of DPPE with two P atoms, TMSPi and trimethyl phosphite (TMPi), with one P atom per molecule as controls, were evaluated using double the contents of TMPi and TMSPi (0.752 ppm) relative to that of DPPE. The respective integrated area ratios of the signals representing HF and PO_2_F_2_^−^ at −193.0 and −85.1 ppm, relative to the internal reference (C_6_F_6_), were compared in different electrolytes. The signals attributed to HF and PO_2_F_2_^−^, which are formed by PF_5_ hydrolysis, are not observed in the presence of DPPE. Conversely, the spectra of the electrolytes with double the amounts of TMPi and TMSPi display the characteristic resonances of HF and PO_2_F_2_^−^, and 52% and 81% of HF are scavenged by TMSPi and TMPi, respectively. In contrast with the case of DPPE, which clearly inhibits PO_2_F_2_^−^ formation, the suppression of PO_2_F_2_^−^ formation is not significant in the presence of TMPi or TMSPi. Clearly, DPPE displays a superior PF_5_‐stabilizing capacity due to the high nucleophilicities of the P atoms compared to those within TMSPi and TMPi, with adjacent highly electronegative O atoms reducing the electron densities of the P atoms. In addition, DFT calculations theoretically confirm that the binding between PF_5_ and the P atoms of DPPE is stronger than that between PF_5_ and the P atoms of TMSPi and TMPi, resulting in superior PF_5_ stabilization (Figure 4c).

Furthermore, the endothermicity and higher activation energy of hydrolysis of PF_5_ coordinated by DPPE indicate that PF_5_ hydrolysis is not favored, in contrast with the reaction without the additive (Figure 4d and Figure S6, Supporting Information). Moreover, DPPE does not generate highly volatile gaseous species, such as TMS‐F with a boiling point of 16 °C, which is formed by the reaction of HF with the TMS group. Further, no color change in the electrolyte with 0.1 wt.% DPPE is observed, whereas the baseline electrolyte exhibits a severe color change to brown after storage for 30 d at 60 °C (Figure S7, Supporting Information). ^19^F NMR spectroscopy confirms that the electrolyte with 0.1 wt.% DPPE stored for 30 d at 60 °C contains less PO_2_F_2_^−^ and HF compared to those in the baseline electrolyte (Figure S8, Supporting Information). Further, the content of HF in 0.1 wt.% DPPE‐containing electrolyte (23 mm) after 100 cycles at 45 °C was lower than baseline electrolyte (51 mm) (Figure S9, Supporting Information). This result demonstrates DPPE effectively stabilizes PF_5_ in electrolyte to suppress hydrolysis reactions generating corrosive HF.

### 2.3 Electrochemical Performances of NCM85/Graphite Full Cells with DPPE

The Ni chelation and PF_5_ stabilization capacities of DPPE were evaluated using Li‐ion cells comprising graphite anodes and NCM85 cathodes with high mass loadings. To avoid errors introduced by the deviation between cells, we evaluated the electrochemical performance of three cells for each electrolyte (Figures S10 and S11, Supporting Information). The full cell with a 0.1 wt.% DPPE‐containing electrolyte exhibits similar capacities to those of the cell with the baseline electrolyte, while delivering a slightly higher initial Coulombic efficiency during precycling (Figure S12a, Supporting Information). This suggests that DPPE does not undergo severe irreversible electrochemical decomposition at the cathode and anode. Moreover, the stabilization of PF_5_ and suppression of HF generation by DPPE lead to the suppression of irreversible reactions (PF_5_ + 2xLi^+^ + 2xe^−^ → xLiF + Li_x_PF_5‐x_, 2HF + 2Li^+^ + 2e^−^ → 2LiF + H_2_),^[^ ^55^ ^]^ which consume Li ions. Thus, the reversible capacity of the NCM85/graphite full cell with DPPE increases with a slightly higher initial Coulombic efficiency relative to that with the baseline electrolyte (Table S5, Supporting Information). In addition, the consumption of DPPE during cycling is negligible, DPPE coordination with Ni^2+^ is observed even after 100 cycles at 45 °C (Figure S13a, Supporting Information), and the content of PO_2_F_2_^−^ decreases (Figure S13b, Supporting Information); these findings are persuasive evidence that DPPE effectively stabilizes PF_5_.

Electrochemical impedance spectroscopy of the full cells after precycling reveals that the cell with the DPPE‐containing electrolyte displays a similar interfacial resistance to that of the cell containing the baseline electrolyte (Figure S12b, Supporting Information). The d*Q*/d*V* plot of the full cell with the DPPE‐containing electrolyte reveals slight shifts in the peaks representing VC and EC reduction at the anode to relatively low potentials during charging, with no additional reduction peaks (Figure S14, Supporting Information). The addition of DPPE leads to the formation of a relatively thinner CEI, as evidenced by the higher relative fractions of the metal—O (from the cathode) peak at 529.9 eV in the O 1s spectra (Figure S15a, Supporting Information) and the C—F (from the PVDF binder) peak at 687.1 eV in the F 1s spectra (Figure S15b, Supporting Information). Further, the graphite anode precycled with the DPPE‐containing electrolyte showed a slightly higher relative fraction of the C—C peak at 284.8 eV compared with that cycled in the baseline electrolyte (Figure S16a, Supporting Information).

**Figure 5:**
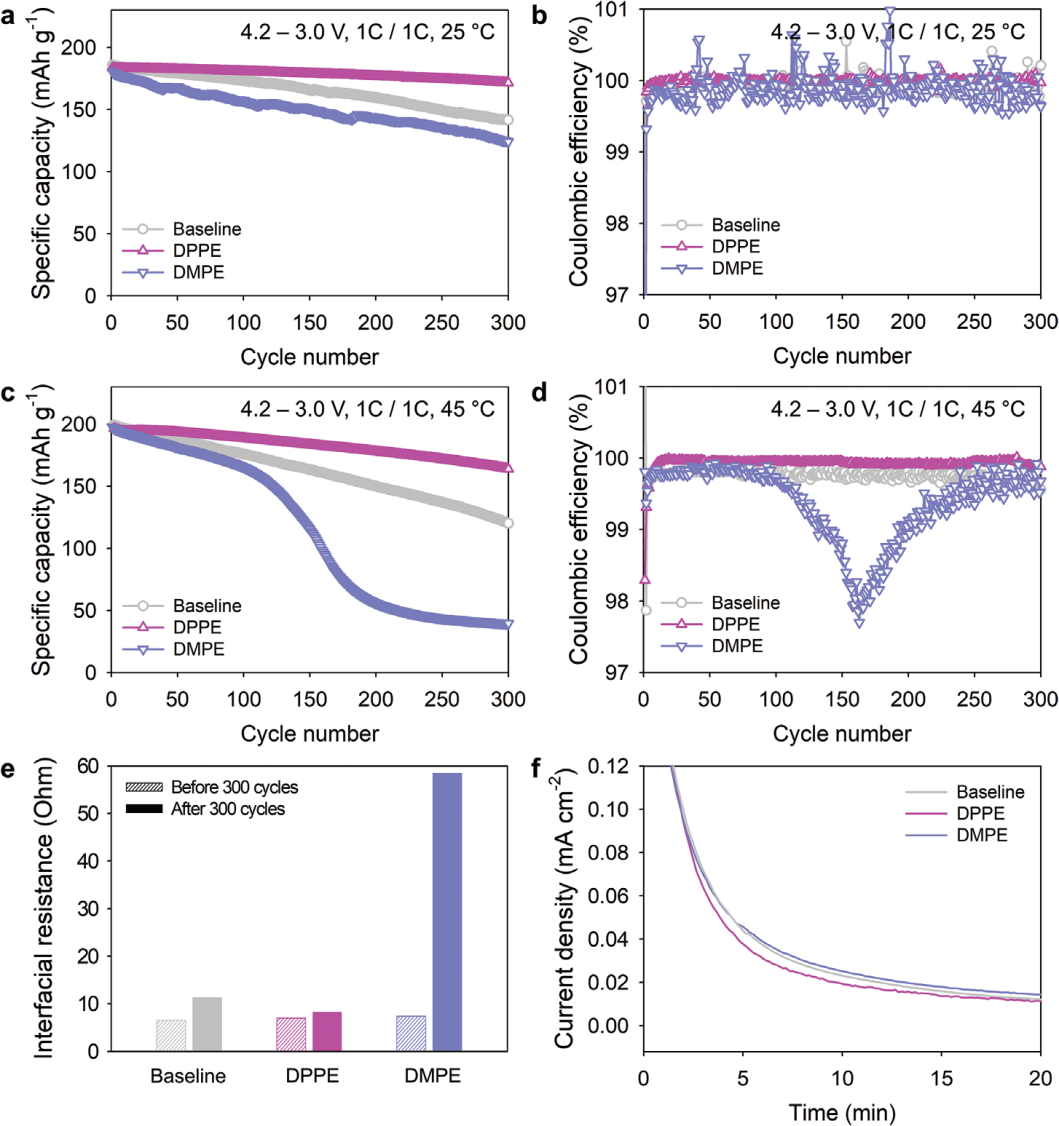
a,c) Cycle performances and b,d) Coulombic efficiency plots of NCM85/graphite full cells at (a, b) 1C and 25 °C and (c, d) 1C and 45 °C with baseline, DPPE‐containing, or DMPE‐containing electrolyte. e) Comparison of the DC‐IR impedances of NCM85/graphite full cells with the DPPE or DMPE additive after 300 cycles at 45 °C. f) Leakage currents of Li/NCM85 half cells with the DPPE or DMPE additive at 4.3 V and 45 °C.

After 300 cycles at 25 °C, the full cell with the DPPE‐containing electrolyte exhibits a drastically improved capacity retention of 93.4% compared to the reduced retention of 76.2% of the cell with the baseline electrolyte (**Figure** **5**a). Notably, the high Coulombic efficiency of the full cell with DPPE was maintained at >99.99% over 300 cycles (Figure 5b).

Moreover, the full cell with DPPE displayed 83.4% of the initial discharge capacity and 99.95% of the initial Coulombic efficiency after 300 cycles at 45 °C, whereas the cell with the baseline electrolyte retains only 60.1% of the initial discharge capacity (Figure 5c,d). Conversely, the full cell with DMPE, which exhibits an immoderate affinity toward Ni ions, had a lower capacity retention compared to the cell with the baseline electrolyte after 300 cycles at 25 and 45 °C (Figure 5a,c). The potential profile of the full cell with the baseline electrolyte cycled at 45 °C reveals a gradual increase in the overpotential, whereas that of the full cell with DPPE is substantially reduced (Figure S17, Supporting Information). This is likely because the addition of DPPE leads to a decrease in the interfacial impedance of the full cell (Figure 5e and Figure S18, Supporting Information). The enhanced cycle performances of the NCM85/graphite full cells are attributed to the Ni^2+^ ion chelation and PF_5_ stabilization of DPPE, which mitigate the interfacial degradation of the electrodes via TM‐DMD. Furthermore, a leakage current study of the NCM85 cathode in a half‐cell configuration at a constant potential of 4.3 V versus Li/Li^+^ after charging at 45 °C reveals that DPPE reduces the leakage current. This indicates the suppression of undesired electrolyte decomposition at the cathode compared to the baseline electrolyte (Figure 5f). This is because DPPE contributes to maintaining the CEI stability via the deactivation of PF_5_, which may generate corrosive HF. DPPE does not adversely affect the charge rate capability of the NCM85/graphite full cell (Figure S19, Supporting Information). To understand the effects of DPPE on the chemical and morphological structures of the CEIs and SEIs on electrodes, the microstructures and chemistry of the NCM85 cathodes and graphite anodes were examined after precycling of the full cells. Cathodes and anodes retrieved from the full cells with DPPE‐containing electrolytes exhibit similar surface morphologies to those of the electrodes precycled in baseline electrolyte, with no discernible decomposition byproducts (Figure S20, Supporting Information). The C 1s X‐ray photoelectron spectra (XPS) of the cathode and anode of the full cell with DPPE reveal the similar intensities of the peaks assigned to C—F due to the polyvinylidene fluoride binder (290.8 eV), Li_2_CO_3_ (290.3 eV), C—O (286.2 eV), and C—C (284.8 eV) to those observed in the spectra of the cathode and anode of the full cell with the baseline electrolyte (Figure S21a,c, Supporting Information).

**Figure 6:**
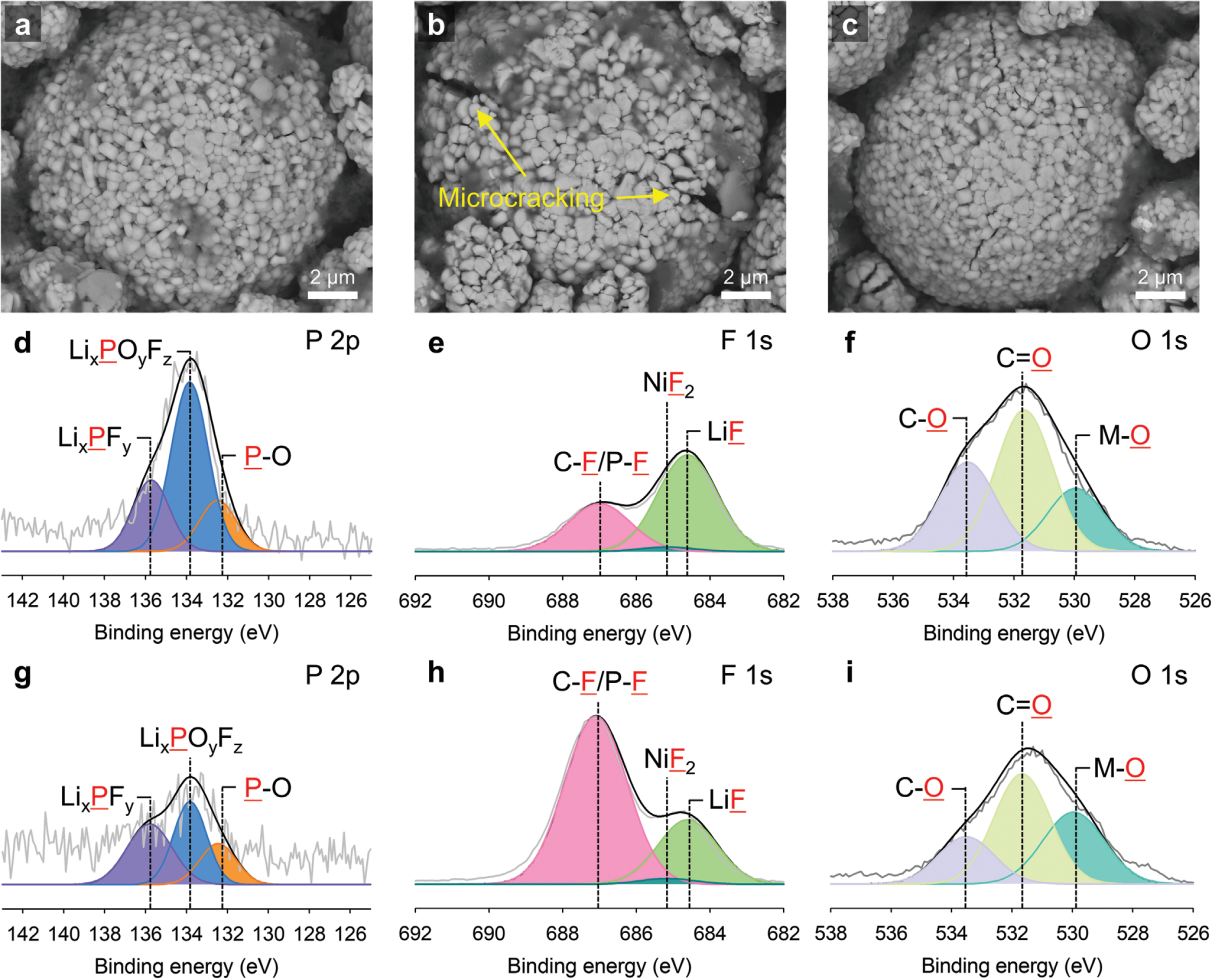
Backscattered‐electron SEM images of the a) pristine NCM85 cathode, NCM85 cathodes with the b) baseline electrolyte or c) DPPE‐containing electrolyte after 300 cycles at 45 °C. d) P 2p, e) F 1s, and f) O 1s XPS spectra of the NCM85 cathode with the baseline electrolyte after 300 cycles at 45 °C. g) P 2p, h) F 1s, and i) O 1s XPS spectra of the NCM85 cathode with the DPPE‐containing electrolyte after 300 cycles at 45 °C.

The spectra of the electrodes removed from the DPPE‐containing electrolyte exhibit peaks representing Li_x_PF_y_ (136.9 eV) and Li_x_PO_y_F_z_ (134.7 eV), but no peak representing P—C (133.0 eV) formed by the decomposition of DPPE is observed (Figure S21b,d, Supporting Information), indicating the electrochemical stability of DPPE in the cell. Based on the XPS spectra, DPPE does not affect the chemical and morphological structures of the CEI and SEI, chelates Ni^2+^ ions in the electrolyte, and coordinates with PF_5_ during cycling. The cathode cycled with the baseline electrolyte at 45 °C showed a surface with electrolyte decomposition byproducts and severe microcracking of the secondary particles **Figure** **6**b). This microcracking is mostly due to the formation of a nonuniform and resistive CEI by the baseline electrolyte, leading to different delithiation levels between the primary particles.^[^ ^56^, ^57^ ^]^ Conversely, the cathode cycled with the DPPE‐containing electrolyte exhibited a clean surface, which is similar to that of a pristine cathode, and small microcracks, which may be formed by the two‐roll pressing process used to prepare the cathode (Figure 6a,c).^[^ ^58^ ^]^ A more homogeneous and less resistive CEI was clearly formed owing to the ability of DPPE to stabilize PF_5_; thus, severe microcracking caused by significant differences in delithiation levels between primary particles did not occur.

The P 2p and F 1s XPS spectra of the cathode cycled with the baseline electrolyte display peaks representing pronounced fractions of LiF (baseline: 64%, DPPE: 26.1%) and Li_x_PO_y_F_z_ (baseline: 58.2%, DPPE: 39.3%, Figure 6d,e,g,h and Tables S6 and S7, Supporting Information). Further, peaks representing strong fractions of C—O (baseline: 30.4%, DPPE: 20%) and C=O (baseline: 48.2%, DPPE: 46.7%) are observed due to increased decomposition of the carbonate solvents, and the intensity of the metal—O peak (baseline: 21.4%, DPPE: 33.3%) decreases owing to a thick CEI blocking the cathode surface (Figure 6f,i and Table S8, Supporting Information). Conversely, the spectra of the cathode cycled with the DPPE‐added electrolyte reveal reductions in the peaks caused by electrolyte decomposition and relatively high fractions of metal—O signals owing to a thin CEI with a high surface coverage. The effect of DPPE on the surface chemistry of the graphite anodes was examined (Figure S22, Supporting Information). The graphite anode cycled with DPPE shows reductions in the intensity of peaks associated with P—F and LiF owing to the PF_5_‐stabilizing effect of DPPE (Figure S22d, Supporting Information). This suppression of PF_5_‐related decomposition leads to a higher relative fraction of C=O species in the VC‐derived SEI, indicating the conservation of this interface (Figure S22c, Supporting Information). Therefore, DPPE, as an electrolyte additive, may stabilize PF_5_, thereby preventing the formation of corrosive HF and helping maintain the quality of the CEI and SEI by restraining undesirable electrolyte decomposition at the surface of the cathode and anode.

### 2.4 Suppression of the Structural Deterioration of the NCM85 Cathode Using DPPE

Structural deterioration from a layered to a rock‐salt phase is a critical limitation^[^ ^59^ ^]^ in the long‐term cycle performances of Ni‐rich cathodes. The layered structure is converted to a spinel‐like and/or rock‐salt structure due to cation mixing via the migration of Ni^2+^, which is formed by the reduction of Ni^4+^ and Ni^3+^, into the Li slab. The migration of Ni^2+^ into this slab, inducing cation mixing, causes the Ni‐rich cathode to lose lattice oxygen, inducing oxygen evolution. The electrochemically inactive rock‐salt phase hinders Li‐ion transport into the bulk structure, resulting in a high cell impedance.^[^ ^60^ ^]^ Comparative X‐ray diffraction (XRD) studies of the NCM85 cathode with and without exposure to DPPE were conducted after 300 cycles at 45 °C (Figure S23 and Table S9, Supporting Information) to confirm the inhibition of cation mixing in the NCM85 cathode by DPPE. The refined XRD data of the pristine cathode and cathodes cycled in the baseline or DPPE‐containing electrolyte reveals that the NCM85 cathode cycled with DPPE suffers less Ni^2+^/Li^+^ cation mixing compared with that cycled with the baseline electrolyte (Figure S23 and Table S10, Supporting Information). The intensity ratio of (003)/(104) (Table S9, Supporting Information), which is the index of cation mixing in the Ni‐rich cathode, is higher upon cycling with the DPPE‐containing electrolyte (1.10) than that observed for the cathode cycled in the baseline electrolyte (1.01).^[^ ^61^ ^]^ The intensity ratio of (003)/(104) of the cathode cycled with the DPPE‐containing electrolyte is closer to the intensity ratio of a pristine cathode (1.18). Hence, the NCM85 cathode cycled with the DPPE‐containing electrolyte undergoes a lower degree of phase transition compared to that of the cathode cycled with the baseline electrolyte. Furthermore, severe peak splitting was observed between the peaks representing (108) and (110) in the XRD pattern of the cathode with the baseline electrolyte, which indicates severe distortion in the hexagonal structure of the layered phase.^[^ ^62^ ^]^

**Figure 7:**
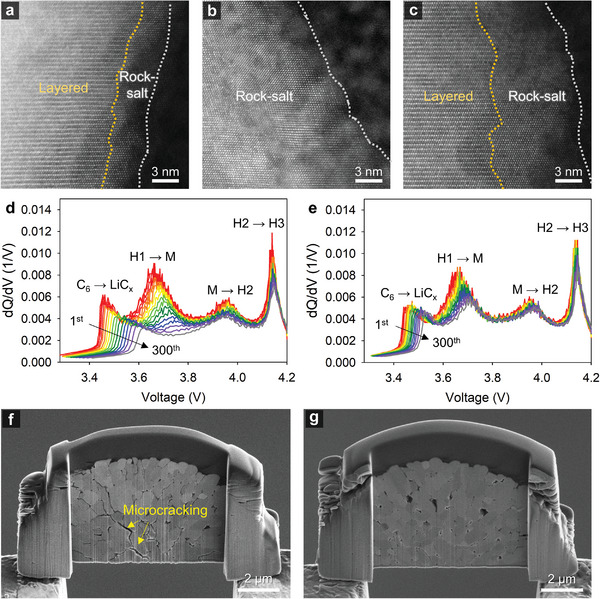
STEM images of the a) pristine NCM85 cathode, b) NCM85 cathode cycled with the baseline electrolyte, and c) NCM85 cathode after 300 cycles at 45 °C with the DPPE‐containing electrolyte. d*Q*/d*V* plots of NCM85/graphite full cells with an increasing number of cycles (from 1 to 300) in the d) baseline or e) DPPE‐containing electrolyte. Cross‐sectional SEM images of the cycled NCM85 cathodes after 300 cycles at 45 °C with the f) baseline or g) DPPE‐containing electrolyte.

**Figure** **7**a shows a scanning transmission electron microscopy (STEM) image of a pristine NCM85 cathode particle with transition metal and Li slabs displayed as bright dotted and dark layers, respectively, in the inner zone and a 3 nm rock‐salt phase, which is observed in Ni‐rich NCM cathodes, on the particle surface.^[^ ^63^, ^64^ ^]^ The NCM85 cathode cycled with the baseline electrolyte at 45 °C showed a thick rock‐salt phase of >14 nm (Figure 7b). In addition, the severely damaged surface of the primary particle owing to the attack of HF was observed in the baseline electrolyte (Figure S24a,b, Supporting Information). Conversely, DPPE drastically reduced the thickness (8.5 nm) of the rock‐salt phase at the cathode surface (Figure 7c), indicating the suppression of structural degradation, which may not ensure the reversibility of lithiation and delithiation.^[^ ^65^ ^]^ Further, DPPE with HF scavenging ability contributed to the preservation of the surface morphology of primary particles (Figure S24c, Supporting Information). This irreversible phenomenon was also observed in the d*Q*/d*V* plots of the charging of NCM85/graphite full cells over 300 cycles at 45 °C. As the number of cycles increases, the peak at 3.65 V attributed to the phase transition from the H1 (hexagonal) phase to the M (monoclinic) phase caused by collective Jahn–Teller distortion of active transition metal ions, such as Ni^3+^ and Mn^3+^,^[^ ^66^, ^67^, ^68^ ^]^ decreased severely for the NCM85 cathode with the baseline electrolyte (Figure 7d). In contrast, the addition of DPPE to the full cell resulted in a smaller decrease in the peak at 3.65 V over 300 cycles (Figure 7e).

Clearly, the structural integrity of the NCM85 cathode remained stable when using the DPPE‐containing electrolyte to prevent the dissolution of transition metal ions. In addition, the peak representing the H2 → H3 phase transition in the d*Q*/d*V* plot at >4.1 V steadily decreased for the NCM85 cathode with the baseline electrolyte. Conversely, the plot of the NCM85 cathode with the DPPE‐containing electrolyte exhibited a peak with an apparently monotonous intensity ascribed to the H2 → H3 phase transition during cycling. Notably, the H2 → H3 phase transition may be reversible if vacant Li slabs with no undesirable occupation of Ni^2+^ in the highly delithiated states, less Ni dissolution, and restrained release of oxygen from the lattice are maintained. Hence, DPPE, with a high Ni chelation capacity, may not forcibly extract Ni ions, which remain in the cathode structure without leaching into the LiPF_6_‐based electrolyte, improving the redox reversibility of the NCM85 cathode during cycling. Notably, the potential corresponding to lithiation of the graphite anode in the full cell with the baseline electrolyte is increased drastically from 3.45 to 3.60 V during cycling, indicating the increase in potential attributed to C_6_ → LiC_x_ (Figure 7d). This may be explained by the consecutive dissolution of transition metal ions from the NCM85 cathode, as initiated by PF_5_ and HF. This causes an increase in the overpotential of Li intercalation within the graphite anode because of the electrodeposited transition metal. Notably, the shift in potential corresponding to C_6_ → LiC_x_ in the full cell with the DPPE‐containing electrolyte is less significant (Figure 7e). This suggests that using DPPE aids in maintaining the high quality of the SEI on the graphite anode, enabling Li‐ion intercalation into the graphite without generating a large overpotential due to the PF_5_ stabilization and Ni chelation of DPPE. Li^+^ removal from the cathode under further delithiation weakens the pillaring effects of the transition metal layers and may cause a phase transition due to the destruction of the cathode structure and a substantial change in the interslab distance.^[^ ^69^ ^]^ The drastic phase transition easily weakens the mechanical strength of the NCM85 cathode, resulting in microcracking of the secondary particles.^[^ ^70^ ^]^ The non‐uniform CEI with a high resistance induces inhomogeneous lithiation and delithiation of the NCM85 primary particles, leading to different degrees of expansion and contraction of each primary particle. Uncontrolled mechanical stress between the primary cathode particles causes the microcracking of the secondary cathode particles (Figure 7f and Figure S25a,c, Supporting Information). Crucially, in the presence of DPPE, microcracking of the NCM85 cathode is suppressed compared to that within the cathode with the baseline electrolyte, as revealed by the cross‐sectional scanning electron microscopy (SEM) images shown in Figure 7g and Figure S25b,d, Supporting Information. This result is attributed to the capacity of DPPE to enhance the structural stability of the NCM85 cathode.

## 3 Conclusion

DPPE, as an electrolyte additive, imparted a remarkable cycling stability on a Li‐ion battery (LIB) composed of an NCM85 cathode and a graphite anode. DPPE chelated Ni^2+^, which may occur in the electrolyte, and blocked the generation of undesirable species, which cause Ni^2+^ dissolution from the NCM85 cathode via the destabilization of PF_5_, which leads to HF generation. With the optimized binding force between Ni^2+^ and DPPE, dissolved Ni^2+^ could be effectively trapped, reducing the overpotential of lithiation of graphite caused by electrodeposited Ni. Severe structural deterioration of the NCM85 cathode, including microcracking and phase transition to the rock‐salt phase, was significantly suppressed using DPPE. The results of this study will contribute to significant advances in the development of electrolyte additives, which may selectively trap transition metal ions dissolved in the electrolyte and eliminate the detrimental substances causing transition metal dissolution, thus realizing high‐energy‐density LIBs.

## 4 Experimental Section

### Electrodes and Electrolytes

For the electrochemical studies of the full cells, NCM85 cathodes and graphite anodes were used. The NCM85 cathodes were prepared by mixing LiNi_0.85_Co_0.1_Mn_0.05_O_2_ active material powder (95 wt.%), the conducting material (2.5 wt.% Super‐P), and the binder (2.5 wt.% PVDF) in 1‐methyl‐2‐pyrrolidinone (99.5%, Sigma‐Aldrich, USA).

The graphite anode was fabricated by mixing graphite powder (96 wt.%) and PVDF (KF9200) binder (4 wt.%). The mass loadings of the NCM85 cathode and graphite anode were 14.0 and 10.4 mg cm^−2^, respectively.

The baseline electrolyte was composed of 1.15 m LiPF_6_ in ethylene carbonate (EC)/dimethyl carbonate (DMC)/ethyl methyl carbonate (EMC) (1:2:2 volume ratio) with 1 wt.% VC (Soulbrain, Republic of Korea) to provide a passivation film on the graphite anode. DPPE (99%) and DMPE (>97%) were purchased from Sigma‐Aldrich. The electrolytes were dehydrated via mixing with CaH_2_, followed by filtration. The H_2_O contents of the electrolytes were then confirmed as <10 ppm via Karl Fischer titration (C20, Mettler Toledo, Columbus, OH, USA).

### Electrochemical Evaluation

The cathode and anode were dried under vacuum at 110 °C for 10 h to reduce their contents of trace H_2_O. A polyethylene membrane with a thickness and porosity of 20 µm and 38% (SK ie technology, Republic of Korea), respectively, was used as a separator. For assembly, the electrolyte (15 µL mAh^−1^) was injected into 2032 coin‐type cells in an Ar‐filled glovebox with H_2_O and O_2_ concentrations of <1.0 ppm. The full cells with NCM85 cathodes and graphite anodes were precycled from 3.0 to 4.2 V with a rate of C/10 using a battery cycler (WBCS3000, WonATech, Republic of Korea) to accumulate the SEIs and CEIs. After precycling, the full cells were subjected to 3 cycles from 3.0 to 4.2 V at a rate of C/5 to stabilize the SEIs and CEIs. Subsequently, cycling studies were conducted at 25 and 45 °C from 3.0 to 4.2 V at a rate of 1C for 300 cycles. NCM85 cathode half‐cell was assembled with an NCM85 cathode and Li metal instead of graphite. This half‐cell underwent a formation cycle from 3.0 to 4.3 V at a rate of C/10 at 25 °C. For the leakage current study, the Li/NCM85 half‐cell was charged to and maintained at 4.3 V for 10 h.

### Characterization

To explore the chelation capacity and PF_5_ stabilizing effect of DPPE, ^31^P and ^19^F NMR spectroscopy (AVANCE 3 HD, 400 MHz, Bruker) were conducted with tetrahydrofuran‐d8 (99.5%, NMR grade, Eurisotop, Saint Aubin, France) as the solvent. To determine the coordination ratio of Ni^2+^ and DPPE, DPPE (2.5, 5.0, or 7.5 mm) was mixed with Ni(TFSI)_2_ in EC:EMC:DMC (1:2:2, volume ratio, 2.5 mm), resulting in Ni^2+^:DPPE equivalent ratios of 1:1, 1:2, and 1:3. These solutions were monitored using ^31^P NMR spectroscopy, with trimethyl phosphate (1 wt.%) added as an internal reference. To demonstrate the PF_5_ stabilizing effect of DPPE, electrolytes with DPPE, TMSPi, or TMPi were stored for 1 d at 25 °C. To quantify the degradation products of LiPF_6_, ^19^F NMR spectroscopy was conducted with C_6_F_6_ (1 wt.%) as an internal reference. To prepare the electrode samples for analysis, electrodes from full cells were rinsed with DMC to remove residual electrolytes. To investigate the morphologies of the electrodes, surface and cross‐sectional SEM (JSM‐6700F, JEOL, Tokyo, Japan) were conducted under an ultrahigh vacuum. The sample for use in cross‐sectional SEM, in particular, was prepared using an ion‐milling system (ArBlade 5000, Hitachi, Japan) with an Ar‐ion beam, and ex situ XPS spectroscopy (K‐Alpha, Thermo Fisher Scientific, USA) was conducted to investigate the interfacial chemistry of the electrodes under a high vacuum. TOF‐SIMS (TOF.SIMS 5, IONTOF, Germany) was conducted in the positive mode to analyze the transition metal ions deposited on the graphite anodes using a Bi^3+^ ion beam (25 keV, 0.34 pA). Transition metal ion dissolution and deposition were measured using ICP‐OES (700‐ES, Agilent Technologies, USA), and the extent of the structural deterioration of the NCM85 cathode was investigated via high‐power XRD (SmartLab, Rigaku, Japan) with Cu K*α* radiation in the 2*θ* range from 10° to 80° and STEM (JEM‐2100F, JEOL) with an acceleration voltage of 200 kV.

### Calculations

In this study, DFT calculations were conducted using the DMol^3^ program^[^ ^71^, ^72^ ^]^ (BIOVIA, USA) to investigate the binding energies and reaction mechanisms. All calculations were spin‐polarized under an implicit solvent environment using the conductor‐like screening model^[^ ^73^ ^]^ with a dielectric constant (*ε*) of 8.85 for EC/EMC/DMC (1:2:2, vol.%, *ε* = 95.3/2.9/3.12) at 25 °C,^[^ ^74^ ^]^ which was calculated using the mixing rule.^[^ ^75^ ^]^ For further information, see the calculation details in the Supporting information.

### Statistical Analysis

Electrochemical tests of full cells were repeated three times.

## Conflict of Interest

The authors declare no conflict of interest.

## Supporting information

Supporting Information

## Data Availability

The data that support the findings of this study are available from the corresponding author upon reasonable request.
