# Supplementary material for: Ni‐Ion‐Chelating Strategy for Mitigating the Deterioration of Li‐Ion Batteries with Nickel‐Rich Cathodes
Source: Adv Sci (Weinh). 2022 Dec 16;10(5):2205918. doi: 10.1002/advs.202205918 (PMC9929120; doi:10.1002/advs.202205918)
Supplement: Supplementary file 1 — Supporting Information [file ADVS-10-2205918-s001.pdf]

ADVANCED SCIENCE
Open Access

Supporting Information

for *Adv. Sci.*, DOI 10.1002/advs.202205918

Ni-Ion-Chelating Strategy for Mitigating the Deterioration of Li-Ion Batteries with Nickel-Rich Cathodes

*Seon Yeong Park, Sewon Park, Hyeong Yong Lim, Moonsu Yoon, Jeong-Hee Choi, Sang Kyu Kwak, Sung You Hong and Nam-Soon Choi**

Supporting Information

Ni-Ion-Chelating Strategy for Mitigating the Deterioration of Li-Ion Batteries with Nickel-Rich Cathodes

*Seon Yeong Park, Sewon Park, Hyeong Yong Lim, Moonsu Yoon, Jeong-Hee Choi, Sang Kyu Kwak, Sung You Hong, and Nam-Soon Choi**

Electrochemical Measurements. Full cells with NCM85 cathodes and graphite anodes were precycled from 3.0 to 4.2 V at a rate of C/10 using a battery cycler (WBCS3000, WonATech, Seoul, Republic of Korea) to form the solid electrolyte interphases (SEIs) and cathode electrolyte interfaces (CEIs). After precycling, the full cells were subjected to 3 cycles from 3.0 to 4.2 V at a rate of C/5 to accumulate SEIs and CEIs with stable structures. To study the charge rate capability, the full cells were cycled with different charge current densities from C/2 to 3C with a fixed discharge current density of C/2 between 4.2 and 3.0 V. NCM85/graphite full cells before and after 300 cycles at 45 °C were charged to state of charge of 50%. Then, the cells were discharged and charged with various currents corresponding to C/10, C/5, C/2, 0.7C, and 1C for 10 sec to explore the impact of electrolytes on the direct current-internal resistance (DC-IR) impedance.

Characterization. The baseline electrolyte and that with 1,2-bis(diphenylphosphino)ethane (DPPE, 0.1 wt.%) were stored at 60 °C for 30 days and images of the different electrolytes were captured after 0, 15, and 30 days. After storage for 30 d, ^{19}F NMR spectroscopy was conducted with each electrolyte (300 μL) using tetrahydrofuran-d_8 as the solvent and C_6F_6 (1 wt.%) as an internal reference. The NCM85 cathodes retrieved from the NCM85/graphite full cells, which were charged to 4.2 V with baseline electrolyte (1.15 M $LiPF_6$ in ethylene carbonate/dimethyl carbonate/ethyl methyl carbonate (1/2/2, v/v/v) + 1 wt.% vinylene

carbonate), were stored in 2 g baseline electrolyte. The electrodes used in the other electrolytes were stored in the corresponding electrolyte, i.e., DMPE or DPPE (0.1 wt.%). The different electrolytes containing the delithiated NCM85 cathodes were stored at 60 °C for 20 h. The electrolytes were then filtered using polytetrafluoroethylene syringe filters with pore sizes of 0.20 μm and analyzed via inductively coupled plasma-optical emission spectroscopy (ICP-OES, 700-ES, Agilent Technologies, Santa Clara, CA, USA).

The content of DPPE required to scavenge Ni^{2+} ions dissolved from the NCM85 cathode is calculated using Equation (S1):

Moles of Ni deposited on the anode after 300 cycles at 45 °C = (154 ppm)*(10.4 mg cm^{-2})*(1.54 cm^2)/(58.7 mg $mmol^{-1}$) = 0.042 μmol (S1)

Because electrolyte to capacity ratio of NCM85/graphite full cells is 15 μL mAh^{-1} and capacity of a coin full cell is 5 mAh, volume of electrolyte used is 75 μL. Density of electrolyte is 1.2 g cm^{-3}. Moles of DPPE are calculated using Equation (S1):

Moles of DPPE in 0.1 wt% DPPE-containing electrolyte = (15 μL mAh^{-1})*(5 mAh)*(1.1 g cm^{-3})*(0.1%)/(398.4 mg $mmol^{-1}$) = 0.207 μmol (S2)

Because Ni^{2+} ion is expected to coordinate with two DPPE molecules, 0.084 μmol of DPPE (0.04% DPPE in electrolyte) is required to effectively scavenge Ni^{2+} ions, which may deposit on the graphite anode during cycling. Thus, it can be thought that 0.1% DPPE content in electrolyte is enough to scavenge Ni^{2+} ions dissolved from NCM85 cathode.

Calculations. Density functional theory calculations were performed to investigate the binding of the additive molecules with PF_5 and mechanisms of hydrolysis. The combination of the Becke three-parameter hybrid functional and the Lee-Yang-Parr correlation functional was employed for the exchange-correlation potential,[1,2] and the van der Waals interactions were corrected using the Tkatchenko-Scheffler method.[3] The double numerical plus

polarization 4.4 basis set was used to describe the atomic orbital basis set with a global orbital cutoff of 4.5 Å. Relativistic core treatments were applied for the core electrons, and the self-consistent field was converged with a convergence criterion of 1.0×10^{-6} Ha. The convergence criteria of geometry optimization were set at 1.0×10^{-5} Ha, 0.002 Ha Å^{-1}, and 0.005 Å for energy, force, and displacement, respectively.

The binding energies between PF_5 and the electrolyte additives are calculated using Equation (S3):

$$\Delta E_{\mathrm{binding}} = E_{\mathrm{additive+PF_5}} - E_{\mathrm{additive}} - E_{\mathrm{PF_5}} \quad \text{(S3)}$$

where $E_{\mathrm{additive+PF_5}}$, E_{additive}, and $E_{\mathrm{PF_5}}$ are the total energy of the additive molecule coordinated to PF_5, the energy of the individual additive, and the energy of PF_5, respectively.

The relative Gibbs free energies (ΔG) were estimated to investigate the reaction mechanism. ΔG at $T =$ 298.15 K is calculated using Equation (S4):

$$\Delta G = \Delta H - T\Delta S = \Delta E + P\Delta V - T\Delta S \approx \Delta E - T\Delta S \quad \text{(S4)}$$

where $H =$ enthalpy, $S =$ entropy, $E =$ energy, and $V =$ volume. As the change in volume (ΔV) is marginal, $P\Delta V$ may be ignored. E and S are calculated in terms of the contributions of translational, rotational, and vibrational motions: $E = E_0 + E_{\mathrm{translational}} + E_{\mathrm{rotation}} + E_{\mathrm{vibration}}$ and $S = S_{\mathrm{translational}} + S_{\mathrm{rotation}} + S_{\mathrm{vibration}}$, respectively, where E_0 is the electronic energy of the ground state.

Linear (LST) and quadratic synchronous transit (QST) methods were used to calculate the reaction pathways of PF_5 hydrolysis with and without the different additives.[4,5] The convergence of the root mean square forces of the atoms was set to 0.002 Ha Å^{-1}.

Table S1. Integrated area ratios of the signals representing $[Ni(DPPE)_2]^{2+}$ normalized by trimethyl phosphate (internal reference) in the ^{31}P NMR spectra of the electrolytes with $Ni(TFSI)_2$ and DPPE in equivalent ratios of 1:1, 1:2, and 1:3

	Integrated area ratio	$Ni(TFSI)_2$:DPPE = 1:1	$Ni(TFSI)_2$:DPPE = 1:2	$Ni(TFSI)_2$:DPPE = 1:3
^{31}P	$[Ni(DPPE)_2]^{2+}$/TMP (internal ref.)	0.055	0.11	0.11

Table S2. Results of ICP-OES of different electrolytes stored for 1 d at 60 °C with delithiated cathodes retrieved from NCM85/graphite full cells

Dissolved transition metal ions [ppm]	Ni^{2+}	Co^{2+}	Mn^{2+}
Baseline	0.83	0.02	0.02
0.1 wt.% DPPE	0.37	N/A	0.02
0.1 wt.% DMPE	3.60	0.11	0.07

Table S3. Results of ICP-OES in terms of transition metals (Ni, Co, Mn) deposited on graphite anodes after 300 cycles at 45 °C with the baseline, DPPE-containing, or DMPE-containing electrolyte

Transition metal deposition [ppm]	Ni	Co	Mn
Baseline	154	0.9	26.6
0.1 wt.% DPPE	62.9	N/A	5.9
0.5 wt.% DPPE	65.9	N/A	4.6
0.1 wt.% DMPE	137	2.0	22.0

Table S4. ICP-OES results of transition metal ions (Ni^{2+}, Co^{2+}, Mn^{2+}) in the electrolytes with and without DPPE after 100 cycles at 45 °C

Transition metal deposition [ppm]	Ni	Co	Mn
Baseline	N/A	N/A	N/A
DPPE	11.2	9.4	8.9

Table S5. Charge capacity, discharge capacity, and initial Coulombic efficiency of NCM85/graphite full cells with the baseline or DPPE-containing electrolyte

	Baseline	0.1% DPPE
Charge capacity (mAh/g)	230.2	230.4
Discharge capacity (mAh/g)	201.5	203.5
Initial coulombic efficiency (%)	87.5	88.3

Table S6. Normalized proportions of the chemical bonds revealed by the P 2p XPS spectra of NCM85 cathodes after 300 cycles at 45 °C with the baseline or DPPE-containing electrolyte

P 2p	136.9 eV Li_xPF_y	134.7 eV $Li_xPO_yF_z$	132.5 eV (P–O)
Baseline	24.4 %	58.2 %	17.4 %
DPPE	39.3 %	39.3 %	21.4 %

Table S7. Normalized proportions of the chemical bonds revealed by the F 1s XPS spectra of NCM85 cathodes after 300 cycles at 45 °C with the baseline or DPPE-containing electrolyte

F 1s	686.9 eV C–F(PVDF), P–F	685.2 eV NiF_2	684.6 eV LiF
Baseline	33.3 %	2.7 %	64 %
DPPE	71.8 %	2.1 %	26.1 %

Table S8. Normalized proportions of the chemical bonds revealed by the O 1s XPS spectra of NCM85 cathodes after 300 cycles at 45 °C with the baseline or DPPE-containing electrolyte

O 1s	533.5 eV<br>C–O	531.6 eV<br>C=O	529.9 eV<br>Metal–O
Baseline	30.4 %	48.2 %	21.4 %
DPPE	20 %	46.7 %	33.3 %

Table S9. Calculated data of the XRD measurements of a pristine cathode and cathodes cycled in the baseline or DPPE-containing electrolyte over 300 cycles at 45 °C

	Peak area of (003)	Peak area of (104)	Ratio of peak intensity of (003)/(104)
Pristine	14622	12386	1.18
Baseline	11544	11388	1.01
DPPE	11894	10717	1.10

Table S10. Refined XRD data of the pristine cathode and cathodes cycled in the baseline or DPPE-containing electrolyte over 300 cycles at 45 °C

Pristine	Element	Site	*x*	*y*	*z*	Occupancy
	Li	3a	0	0	0	0.987(6)
a = 2.874404(3) Å	Li	3b	0	0	0.5	0.013(7)
c = 14.21342(5) Å	Co	3b	0	0	0.5	0.100(4)
R_{wp} = 9.45%	Ni	3b	0	0	0.5	0.837(3)
R_p = 6.58%	Mn	3b	0	0	0.5	0.100(6)
	Ni	3a	0	0	0	0.013(6)
	O	6c	0	0	0.259152	1.0(4)
**Baseline**	**Element**	**Site**	***x***	***y***	***z***	**Occupancy**
	Li	3a	0	0	0	0.814(6)
a = 2.84181(3) Å	Li	3b	0	0	0.5	0.186(6)
c = 14.3991(6) Å	Co	3b	0	0	0.5	0.102(4)
R_{wp} = 10.43%	Ni	3b	0	0	0.5	0.664(4)
R_p = 6.29%	Mn	3b	0	0	0.5	0.100(4)
	Ni	3a	0	0	0	0.186(6)
	O	6c	0	0	0.241033	1.0(4)
**DPPE**	**Element**	**Site**	***x***	***y***	***z***	**Occupancy**
	Li	3a	0	0	0	0.893(6)
a = 2.85023(3) Å	Li	3b	0	0	0.5	0.107(6)
c = 14.3420(6) Å	Co	3b	0	0	0.5	0.100(4)
R_{wp} = 10.34%	Ni	3b	0	0	0.5	0.743(4)
R_p = 5.96%	Mn	3b	0	0	0.5	0.100(4)
	Ni	3a	0	0	0	0.107(6)
	O	6c	0	0	0.256735	1.0(4)

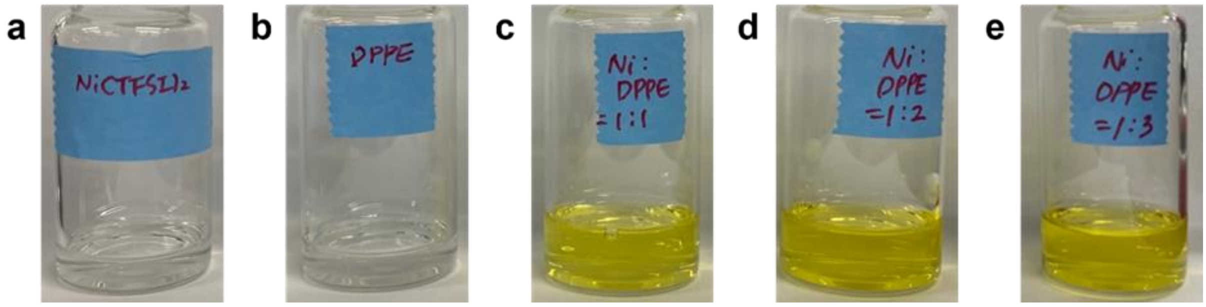

Figure S1. Images of EC/DMC/EMC (1/2/2, v/v/v) solutions with a) 2.5 mM $Ni(TFSI)_2$, b) 2.5 mM DPPE, c) 2.5 mM $Ni(TFSI)_2$ and 2.5 mM DPPE, d) 2.5 mM $Ni(TFSI)_2$ and 5.0 mM DPPE, and e) 2.5 mM $Ni(TFSI)_2$ and 7.5 mM DPPE at 25 °C.

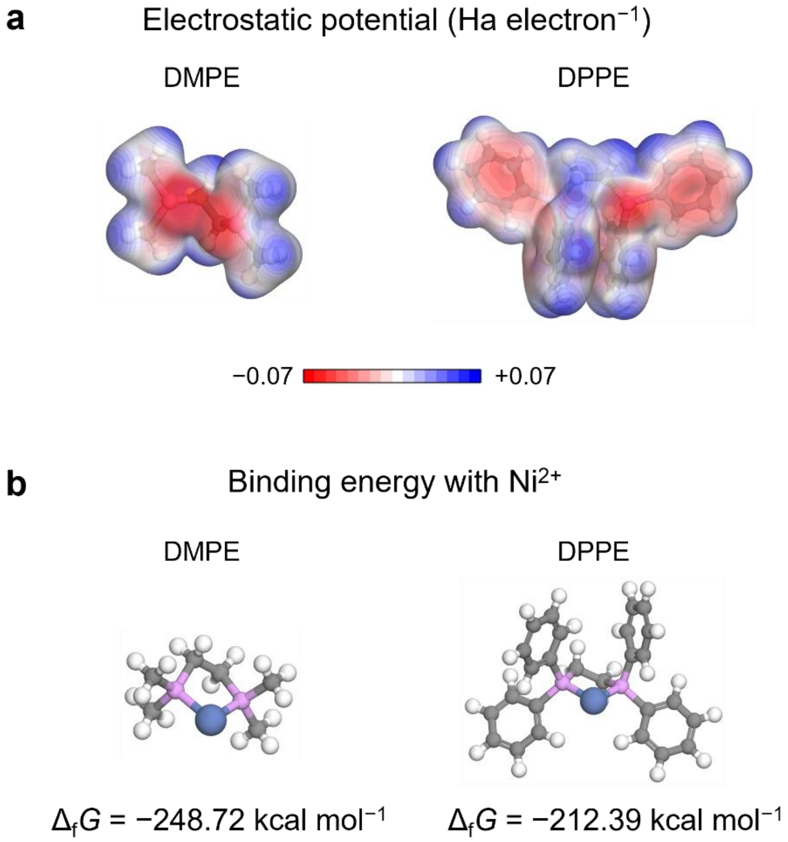

Figure S2. a) Molecular electrostatic potential mapping of DMPE and DPPE. b) Binding energies between DMPE and Ni^{2+}, and between DPPE and Ni^{2+}.

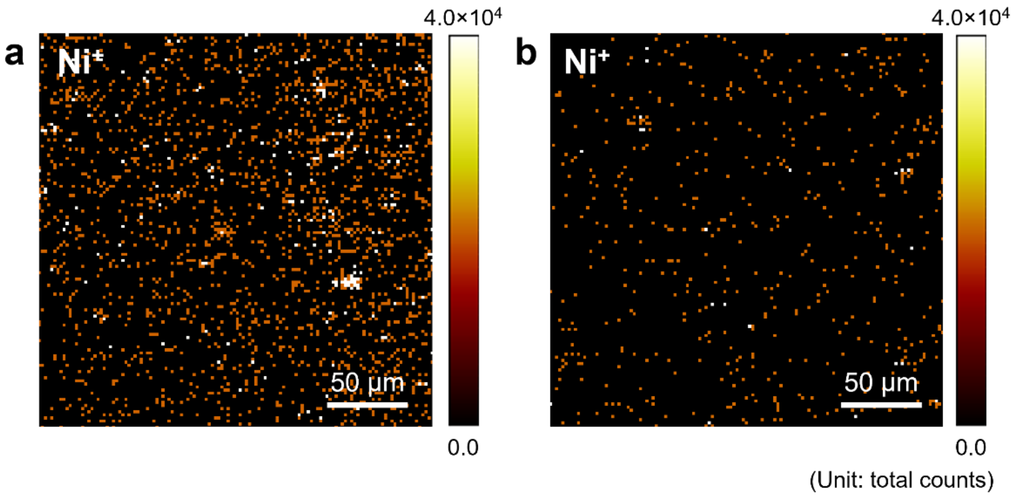

Figure S3. TOF-SIMS images of Ni^{+} on graphite anodes retrieved from full cells with electrolytes containing a) 0.188 mM $Ni(TFSI)_2$ or b) 0.188 mM $Ni(TFSI)_2$ + 0.376 mM DPPE.

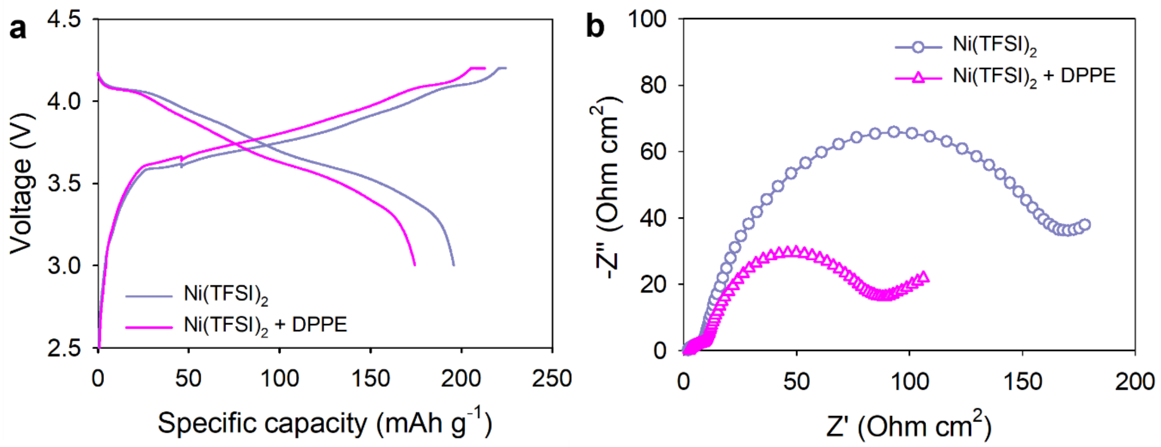

Figure S4. a) Potential profiles of NCM85/graphite full cells with electrolytes containing 0.188 mM $Ni(TFSI)_2$ or 0.188 mM $Ni(TFSI)_2$ + 0.376 mM DPPE during precycling. b) Nyquist plots of NCM85/graphite full cells with electrolytes containing 0.188 mM $Ni(TFSI)_2$ or 0.188 mM $Ni(TFSI)_2$ + 0.376 mM DPPE after precycling.

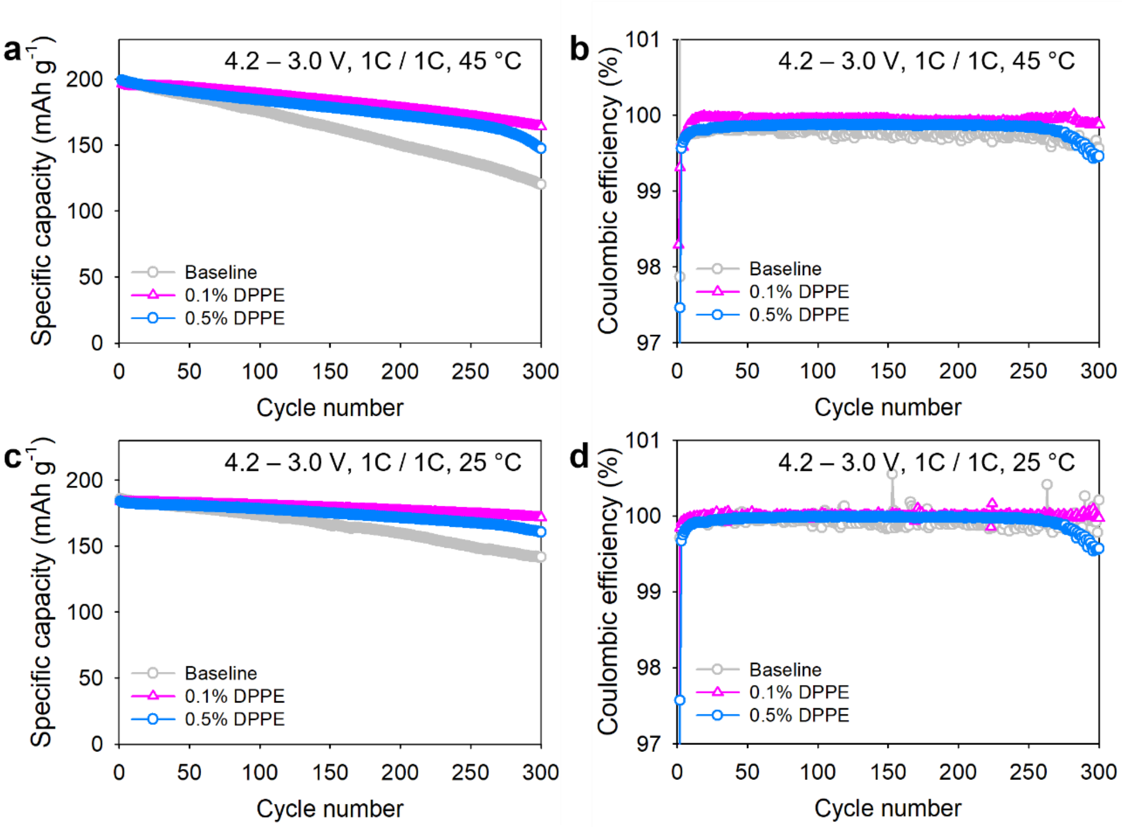

Figure S5. a, c) Cycling performance and (b, d) Coulombic efficiency plots of NCM85/graphite full cells at a, b) 45 °C and c, d) 25 °C with the baseline, 0.1% DPPE-containing, or 0.5% DPPE-containing electrolyte.

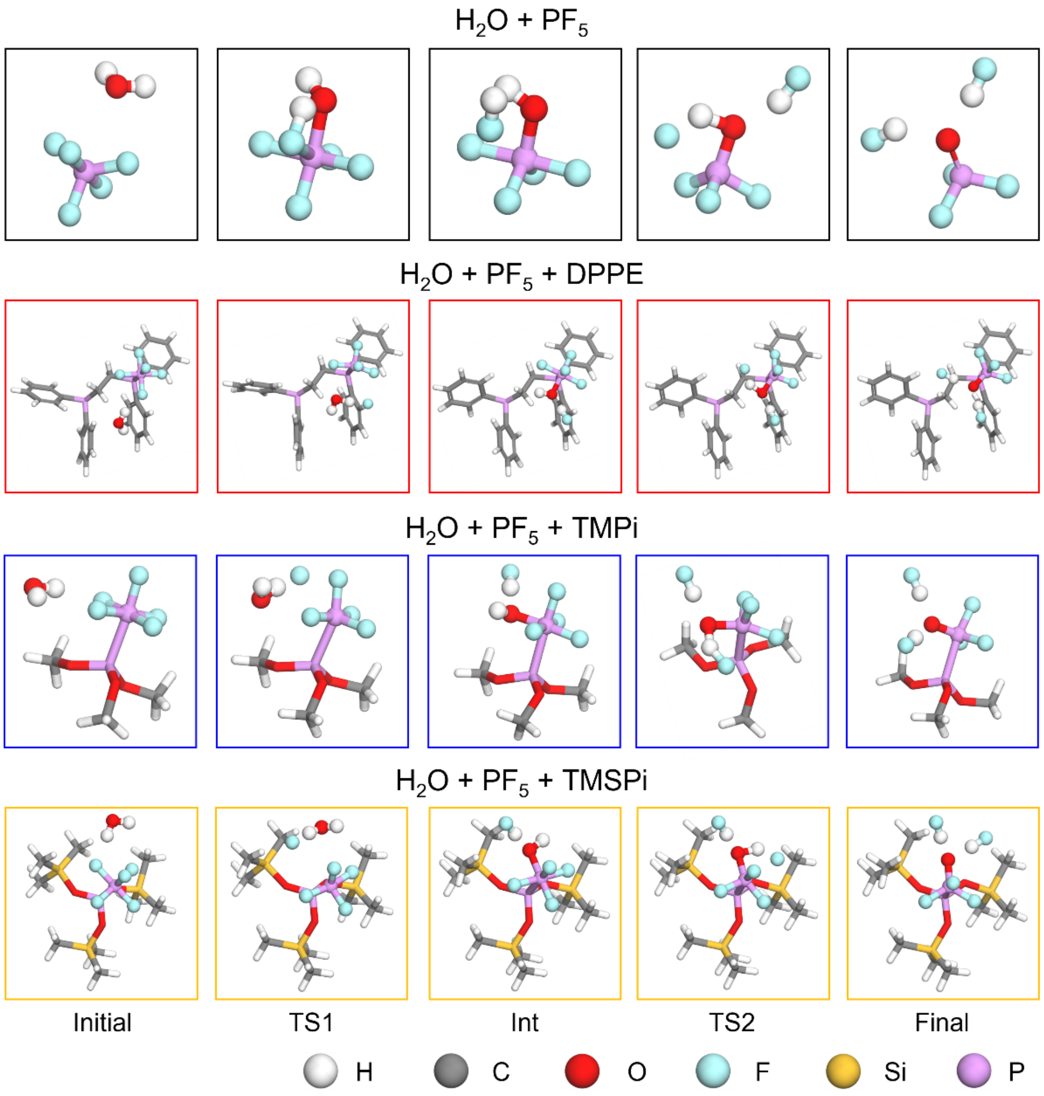

Figure S6. Molecular configurations in the hydrolysis of PF_5 with and without different additives. TS1: transition state 1, Int: intermediate step, TS2: transition state 2.

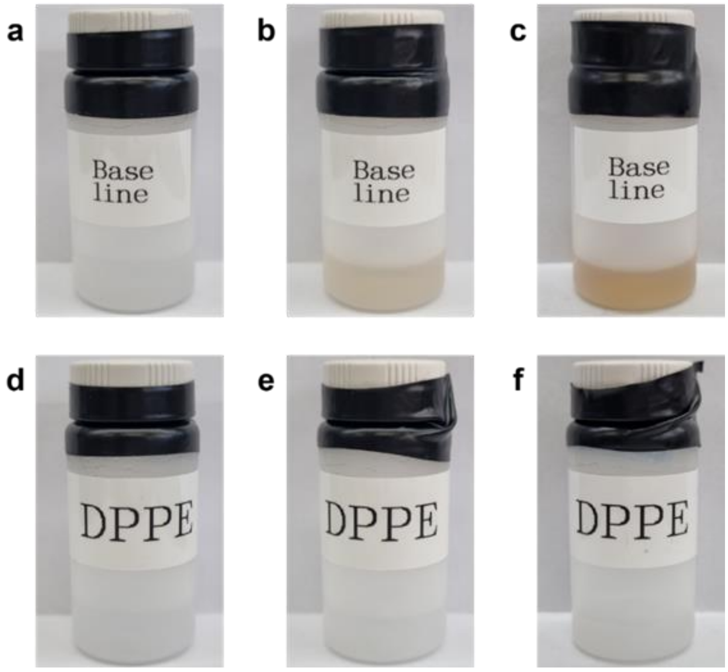

Figure S7. Images of the baseline electrolyte stored at 60 °C for a) 0, b) 15, and c) 30 d and the DPPE-containing electrolyte stored at 60 °C for d) 0, e) 15, and f) 30 d.

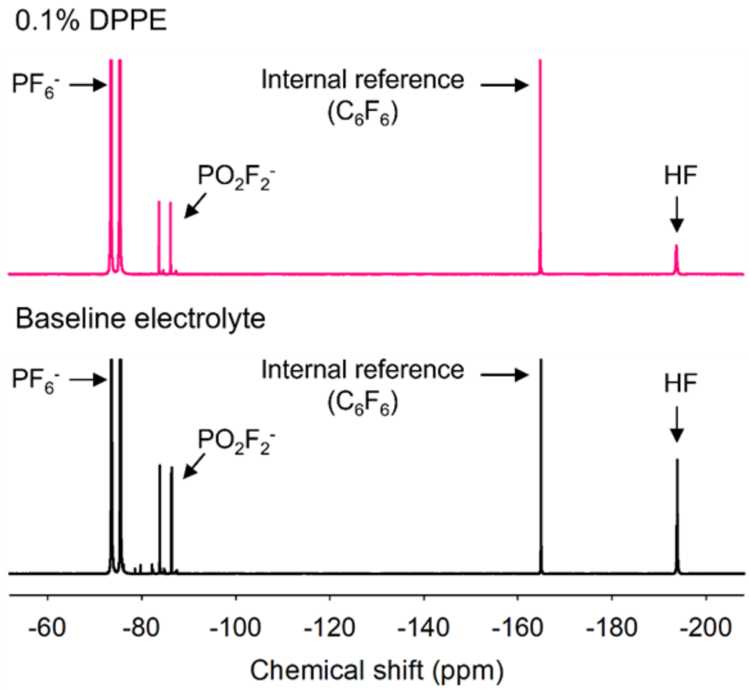

Figure S8. ^{19}F NMR spectra of electrolytes stored for 30 d at 60 °C with and without 0.1 wt.% DPPE.

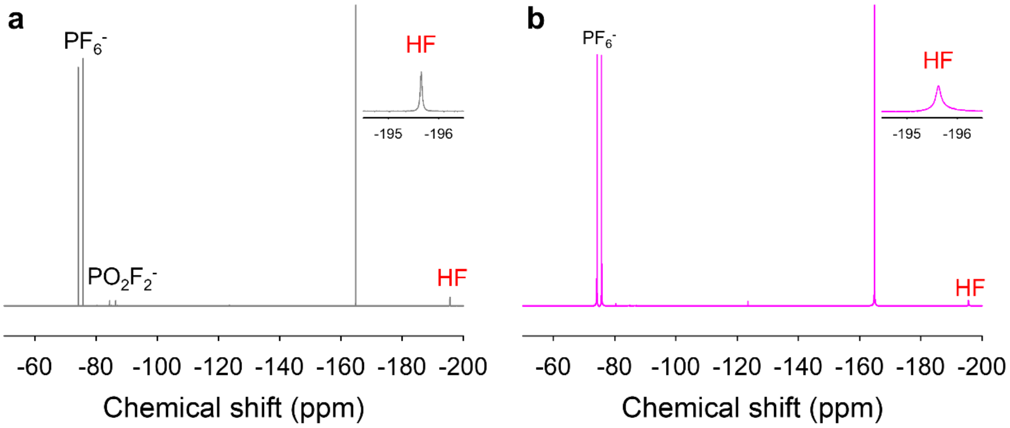

Figure S9. ^{19}F NMR spectra of the a) baseline electrolyte and b) DPPE-containing electrolyte retrieved from NCM85/graphite full cells after 100 cycles at 45 °C.

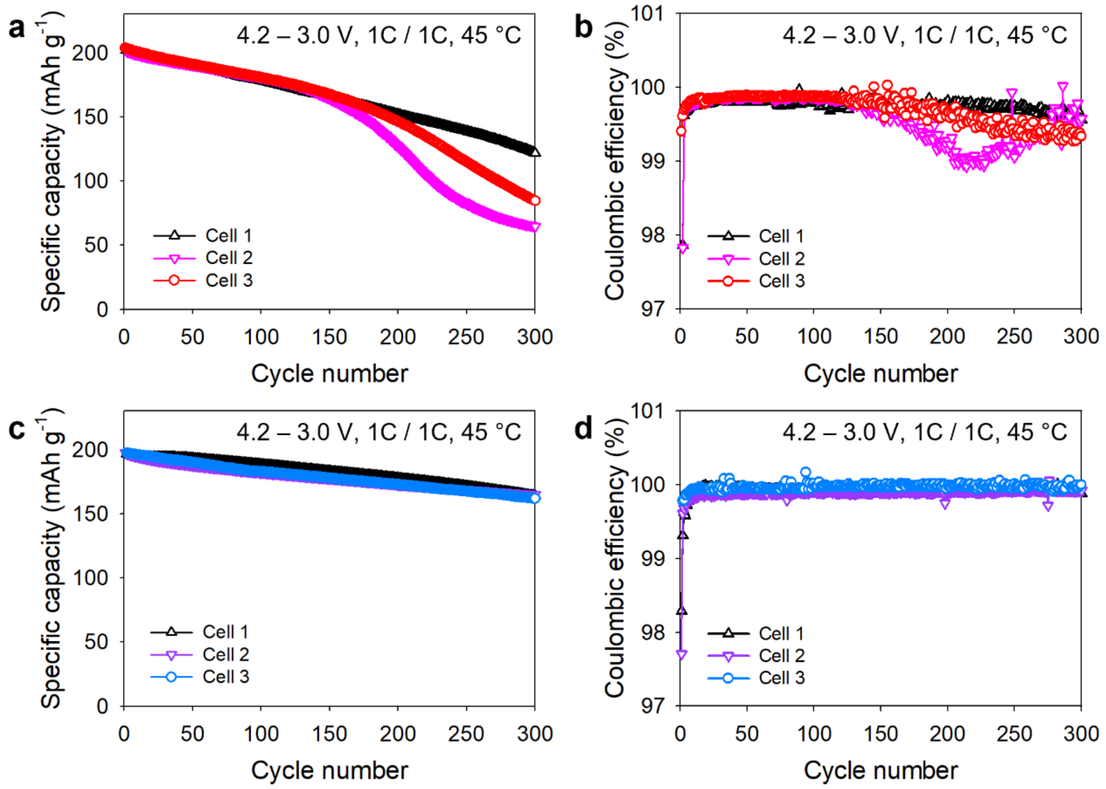

Figure S10. a, c) Cycling performance and b, d) Coulombic efficiency plots of the NCM85/graphite full cells at 45 °C with the a, b) baseline electrolyte and c, d) DPPE-containing electrolyte.

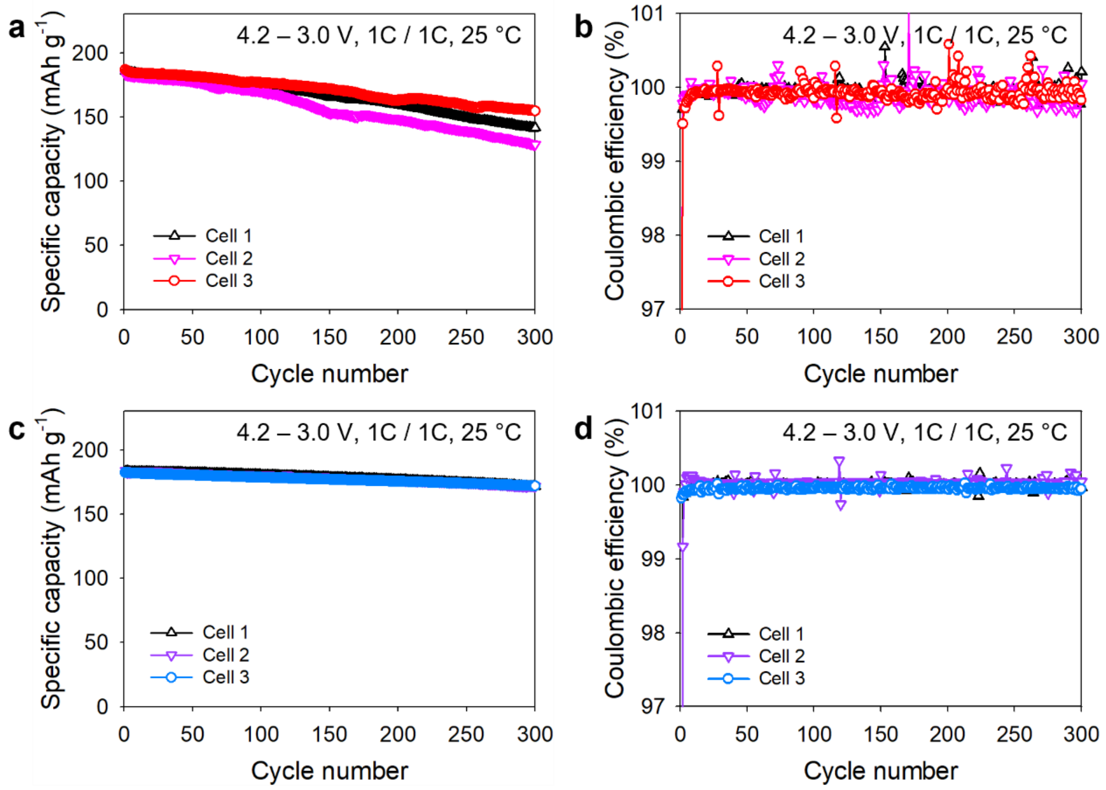

Figure S11. a, c) Cycling performance and b, d) Coulombic efficiency plots of the NCM85/graphite full cells at 25 °C with the a, b) baseline electrolyte and c, d) DPPE-containing electrolyte.

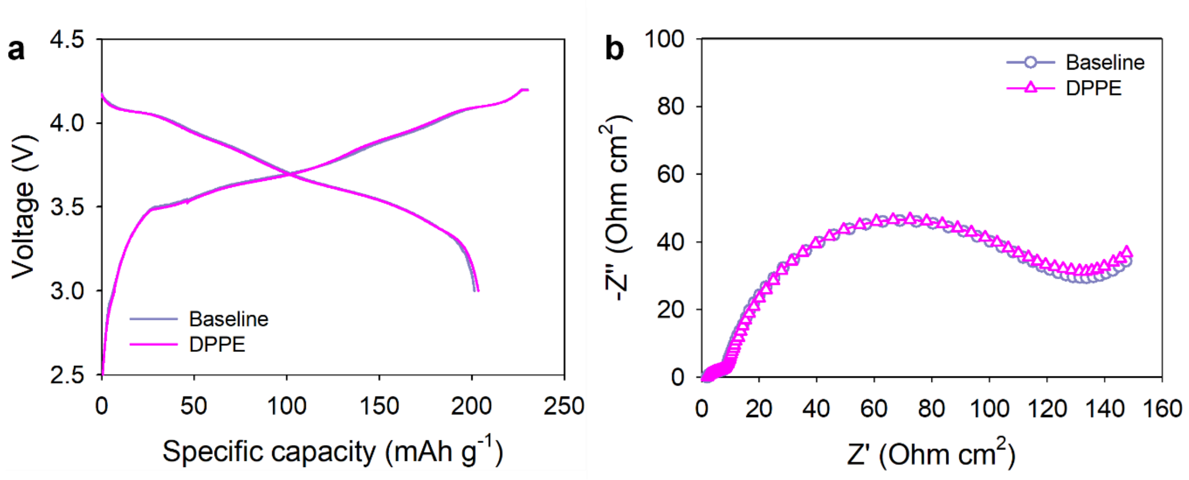

Figure S12. a) Charge-discharge profiles of NCM85/graphite full cells with the baseline or DPPE-containing electrolyte during precycling. b) Nyquist plots of NCM85/graphite full cells with the baseline or DPPE-containing electrolyte after precycling.

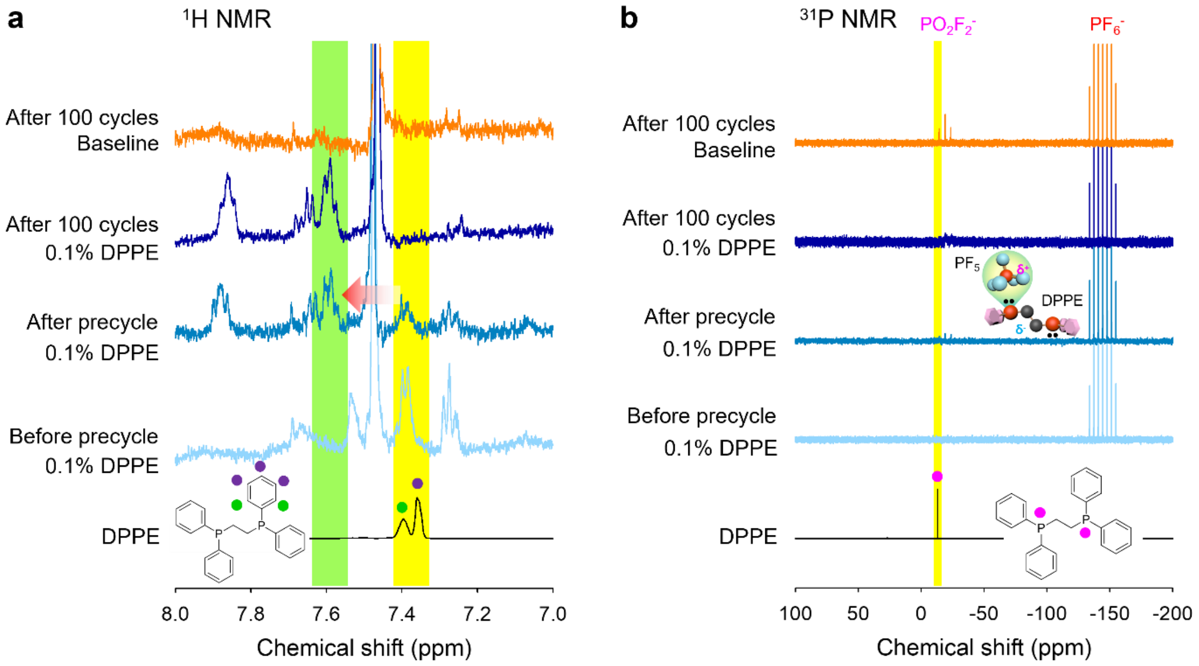

Figure S13. a) ^{1}H NMR and b) ^{31}P NMR spectra of the electrolytes retrieved from the NCM85/graphite full cells after cell assembly (before cycling), after precycling, and after 100 cycles at 45 °C. The peak intensities in the ^{31}P NMR spectra were normalized by the PF_6^- peak at −146.1 ppm and the peak intensities in the ^{1}H NMR spectra were normalized by the ethylene carbonate peak at 4.54 ppm.

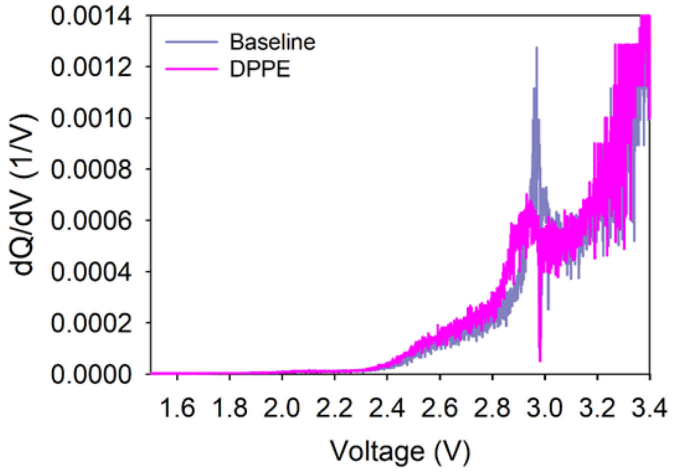

Figure S14. dQ/dV plots of NCM85 cathode/graphite full cells with the baseline or DPPE-containing electrolyte during precycling.

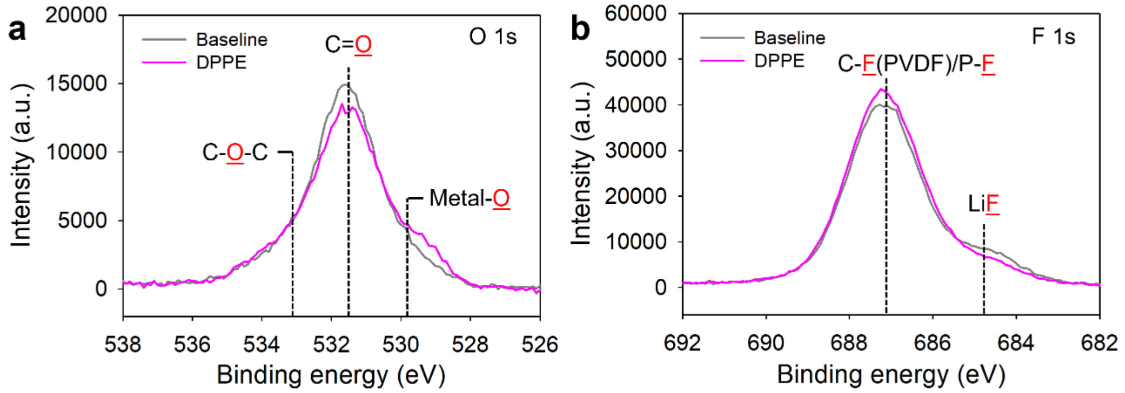

Figure S15. a) O 1s and b) F 1s spectra of NCM85 cathodes after precycling with the baseline or DPPE-containing electrolyte.

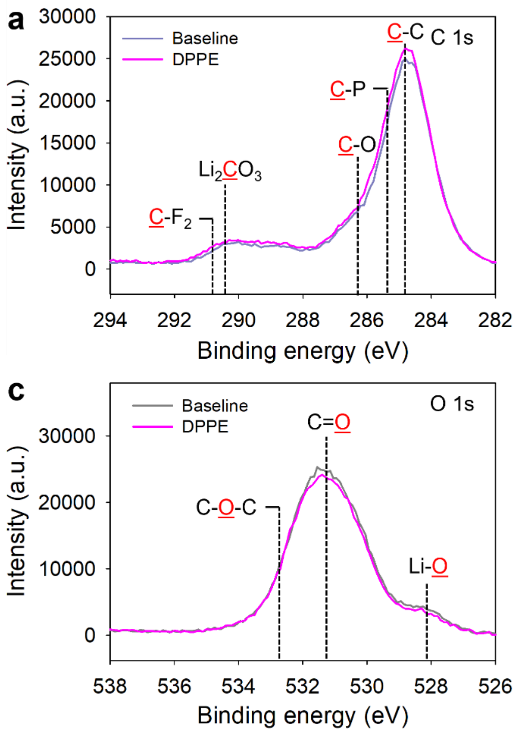

Figure S16. a) C 1s, b) P 2p, c) O 1s, and d) F 1s spectra of graphite anodes after precycling with the baseline or DPPE-containing electrolyte.

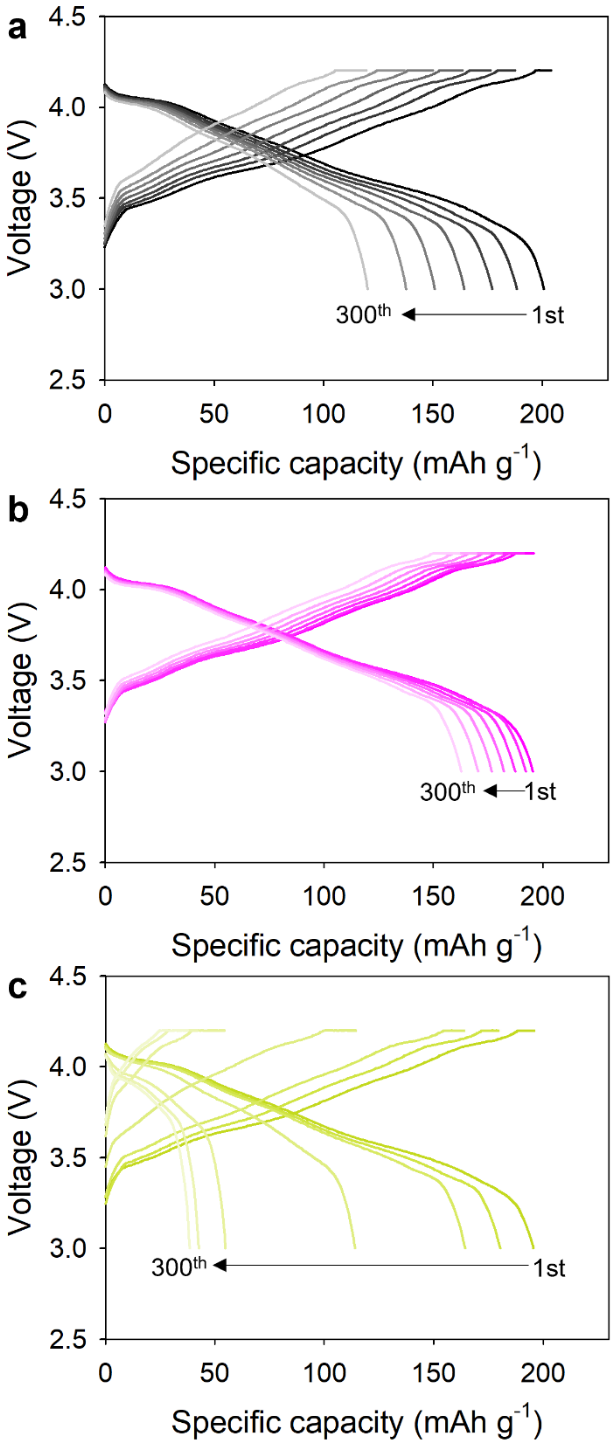

Figure S17. Potential profiles of NCM85/graphite full cells with the a) baseline electrolyte, b) DPPE-containing electrolyte, or c) DMPE-containing electrolyte with an increasing number of cycles (from 1 to 300).

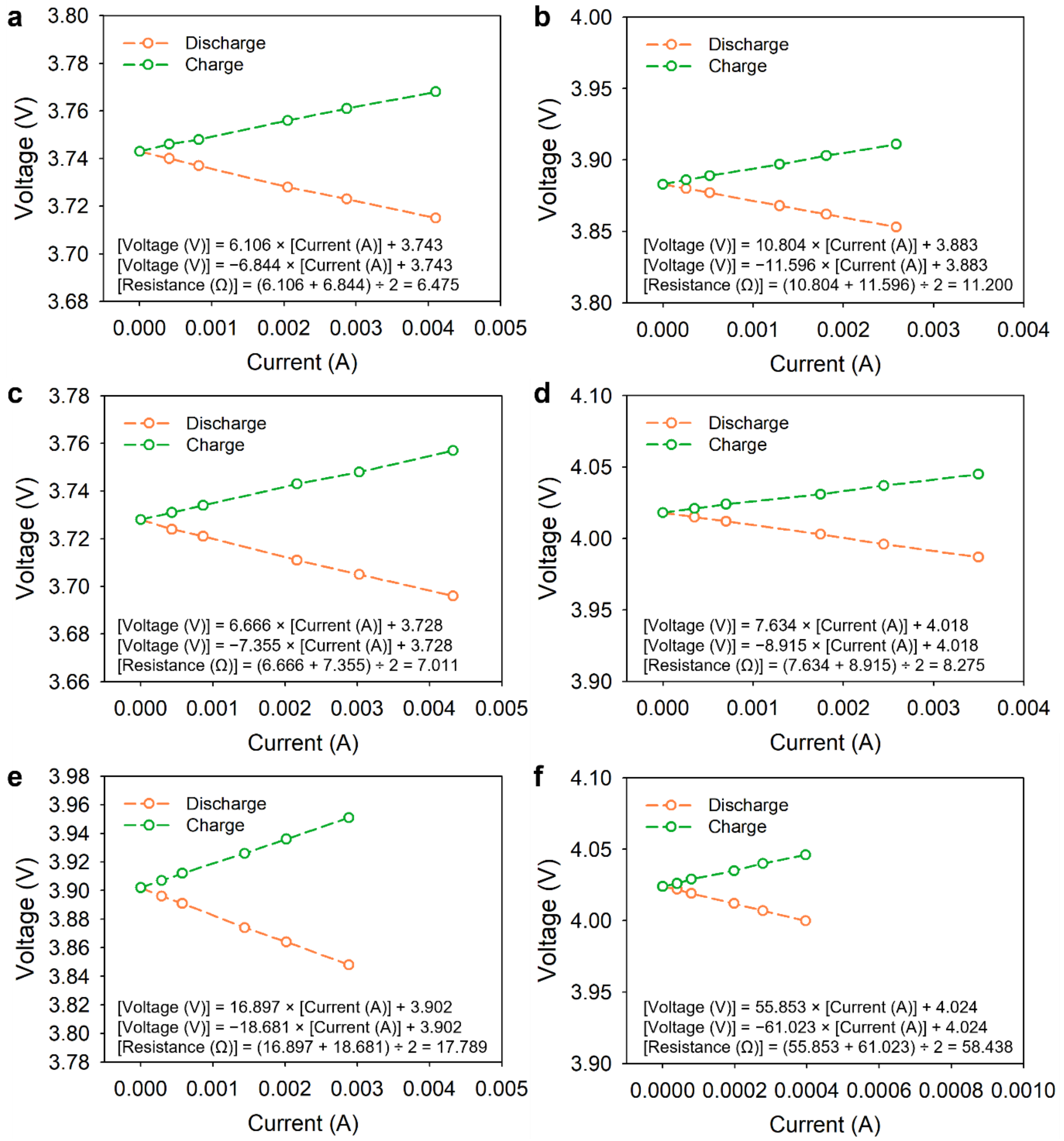

Figure S18. DC-IR tests of NCM85/graphite full cells before cycling at 45 °C with the a) baseline electrolyte, b) DPPE-containing electrolyte, or c) DMPE-containing electrolyte and after 300 cycles at 45 °C with the b) baseline electrolyte, d) DPPE-containing, or f) DMPE-containing electrolyte.

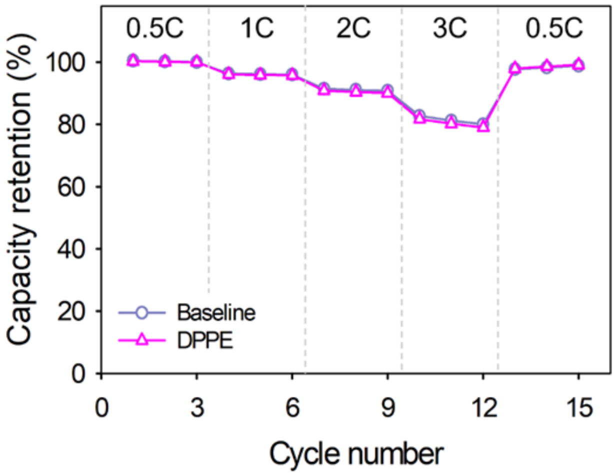

Figure S19. Charge rate capabilities of NCM85/graphite full cells at a fixed discharge rate of 0.5 C.

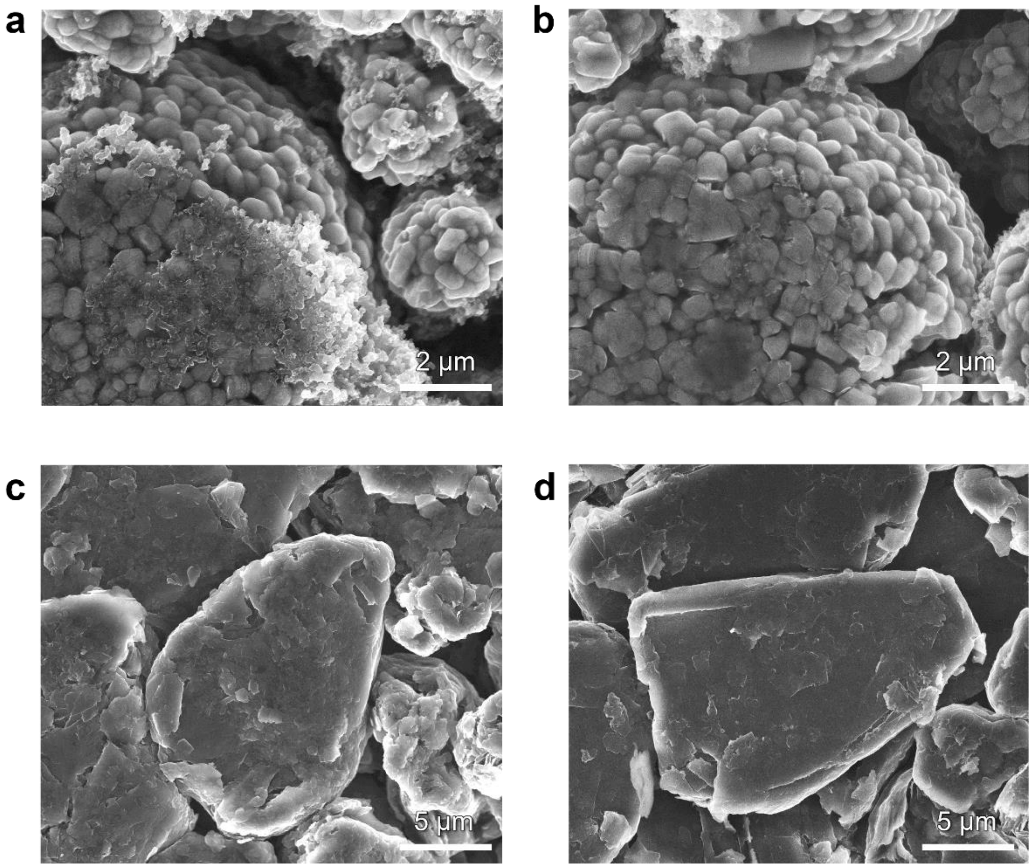

Figure S20. Top-view SEM images of NCM85 cathodes after precycling with the a) baseline or b) DPPE-containing electrolyte. Top-view SEM images of graphite anodes after precycling with the c) baseline or d) DPPE-containing electrolyte.

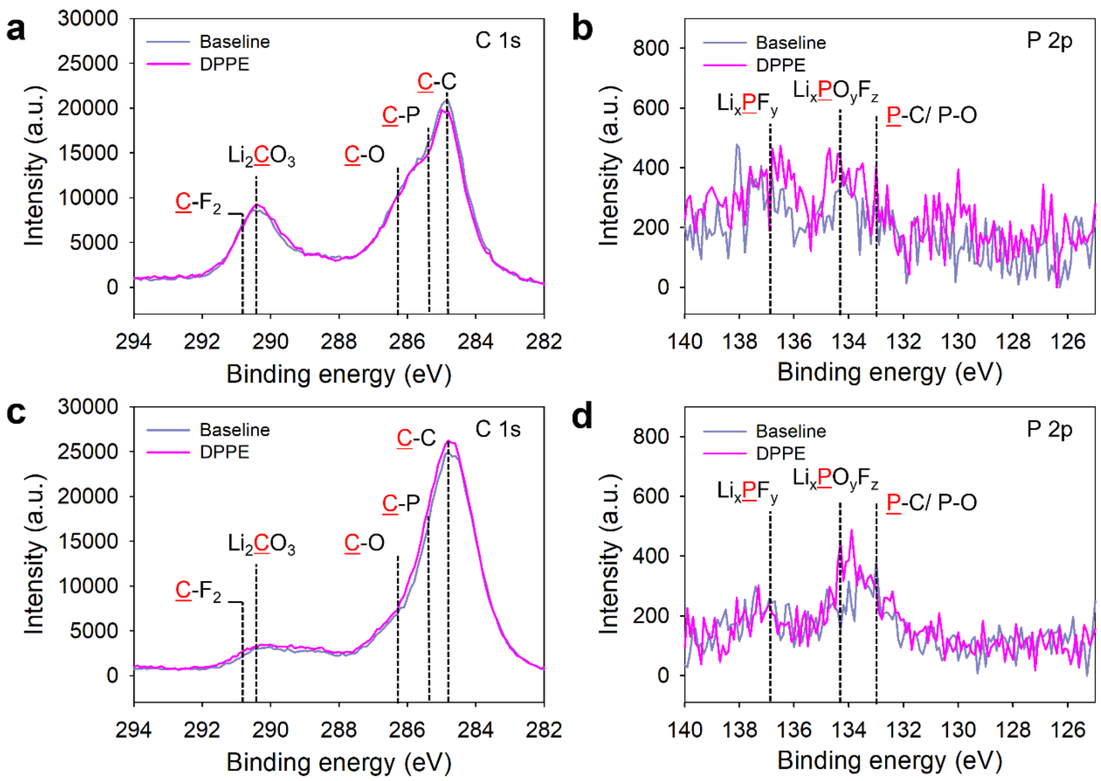

Figure S21. a) C 1s and b) P 2p spectra of NCM85 cathodes after precycling with and without exposure to DPPE. c) C 1s and d) P 2p XPS spectra of the graphite anodes after precycling with and without exposure to DPPE.

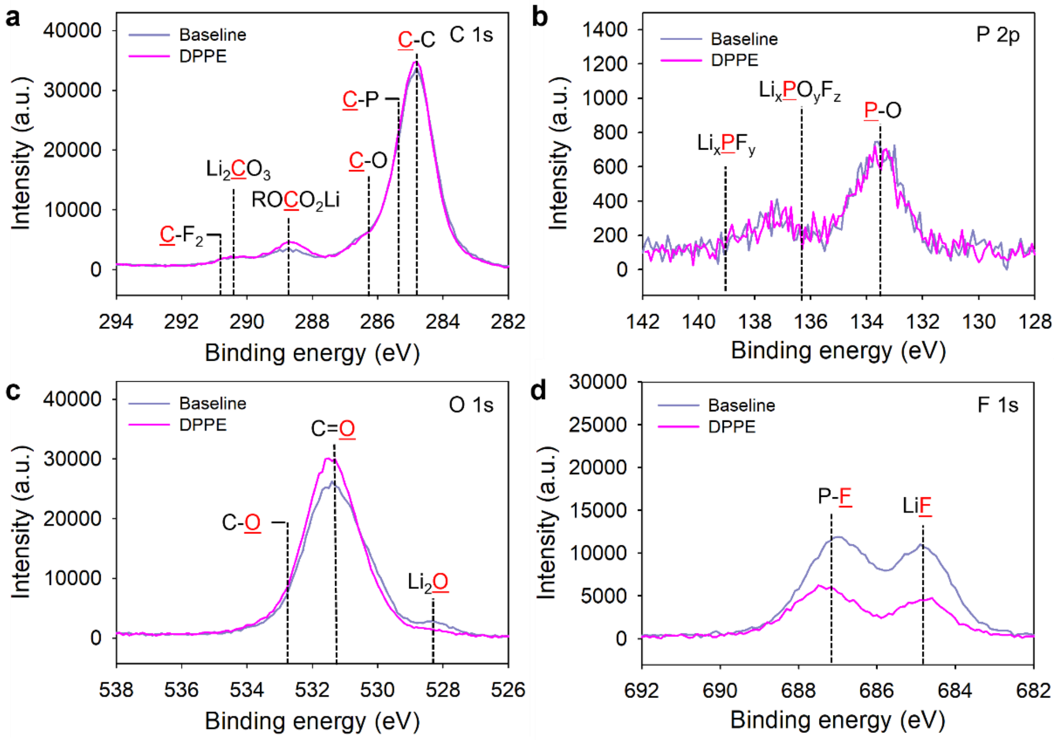

Figure S22. a) C 1s, b) P 2p, c) P 2p, and d) F 1s spectra of graphite anodes with the baseline or DPPE-containing electrolyte after 300 cycles at 45 °C.

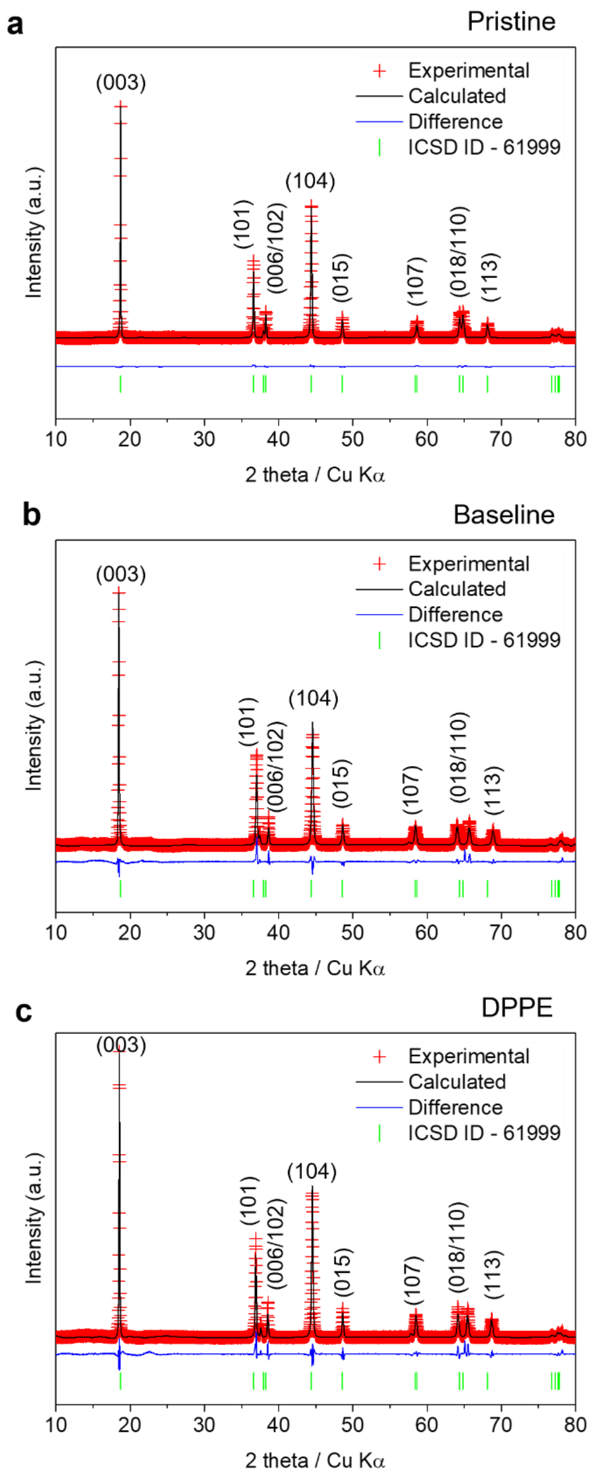

Figure S23. Rietveld refinement results of the XRD patterns of the (a) pristine NCM85 cathode, (b) NCM85 cathode cycled with the baseline electrolyte, and (c) NCM85 cathode cycled with the DPPE-containing electrolyte after 300 cycles at 45 °C.

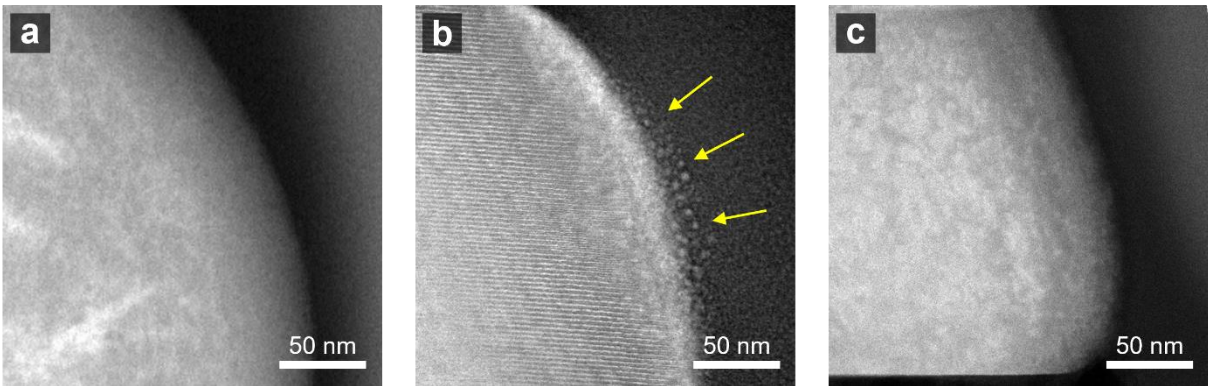

Figure S24. STEM images of the a) pristine NCM85 cathode, b) NCM85 cathode cycled with the baseline electrolyte, and c) NCM85 cathode cycled with the DPPE-containing electrolyte after 300 cycles at 45 °C.

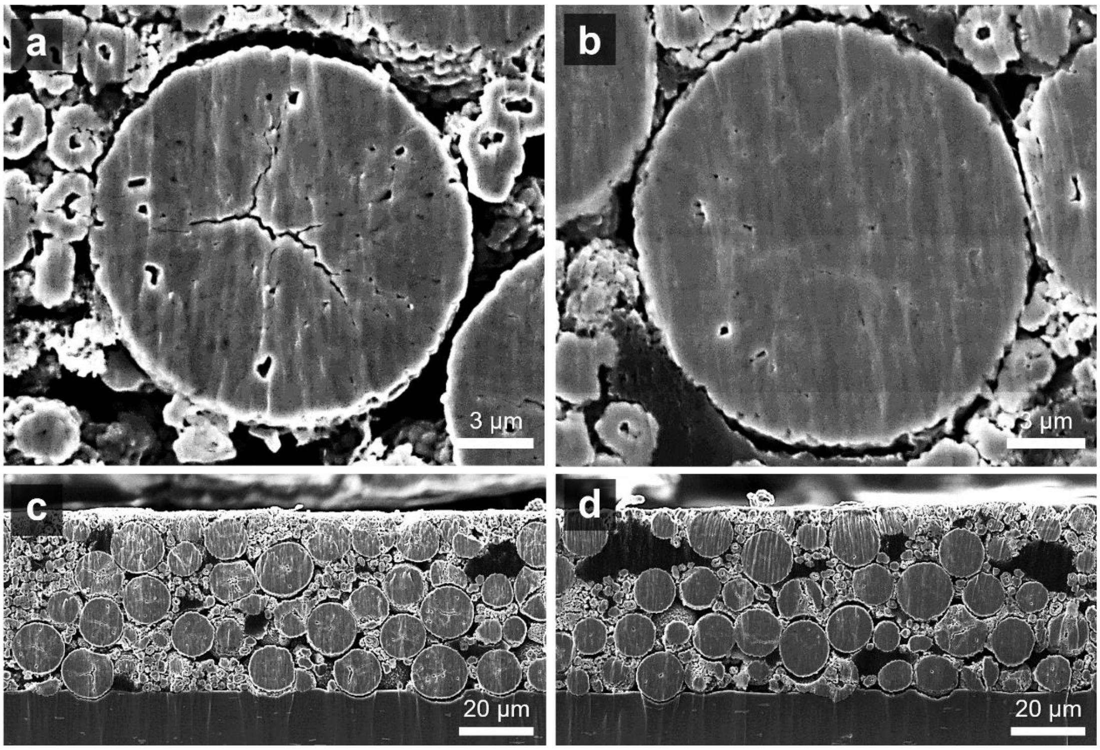

Figure S25. Cross-sectional SEM images of NCM85 cathodes cycled with the a,c) baseline or b,d) DPPE-containing electrolyte after 300 cycles at 45 °C.

References

[1] A. D. Becke, *J. Chem. Phys.* **1993**, *98,* 5648.

[2] P. J. Stephens, F. J. Devlin, C. F. Chabalowski, M. J. Frisch, *J. Phys. Chem.* **1994**, *98*, 11623.

[3] A. Tkatchenko, M. Scheffler, *Phys. Rev. Lett.* **2009**, *102*, 073005.

[4] T. A. Halgren, W. N. Lipscomb, *Chem. Phys. Lett.* **1977**, *49*, 225.

[5] N. Govind, M. Petersen, G. Fitzgerald, D. King-Smith, J. Andzelm, *Comput. Mater. Sci.* **2003**, *28*, 250.
